# Marine biodiversity and the chessboard of life

**DOI:** 10.1371/journal.pone.0194006

**Published:** 2018-03-22

**Authors:** Grégory Beaugrand, Christophe Luczak, Eric Goberville, Richard R. Kirby

**Affiliations:** 1 CNRS, Univ. Lille, Univ. Littoral Côte d’Opale, UMR 8187, LOG, Laboratoire d’Océanologie et de Géosciences, Wimereux, France; 2 Sir Alister Hardy Foundation for Ocean Science, The Laboratory, Citadel Hill, Plymouth, United Kingdom; 3 Université d’Artois, ESPE, Centre de Gravelines, Gravelines, France; 4 The Secchi Disk Foundation, Kiln Cottage, Gnaton, Yealmpton, United Kingdom; University of Sydney, AUSTRALIA

## Abstract

Species richness is greater in places where the number of potential niches is high. Consequently, the niche may be fundamental for understanding the arrangement of life and especially, the establishment and maintenance of the well-known Latitudinal Biodiversity Gradient (LBG). However, not all potential niches may be occupied fully in a habitat, as measured by niche vacancy/saturation. Here, we theoretically reconstruct oceanic biodiversity and analyse modeled and observed data together to examine patterns in niche saturation (i.e. the ratio between observed and theoretical biodiversity of a given taxon) for several taxonomic groups. Our results led us to hypothesize that the arrangement of marine life is constrained by the distribution of the maximal number of species’ niches available, which represents a fundamental mathematical limit to the number of species that can co-exist locally. We liken this arrangement to a type of chessboard where each square on the board is a geographic area, itself comprising a distinct number of sub-squares (species’ niches). Each sub-square on the chessboard can accept a unique species of a given ecological guild, whose occurrence is determined by speciation/extinction. Because of the interaction between the thermal niche and changes in temperature, our study shows that the chessboard has more sub-squares at mid-latitudes and we suggest that many clades should exhibit a LBG because their probability of emergence should be higher in the tropics where more niches are available. Our work reveals that each taxonomic group has its own unique chessboard and that global niche saturation increases when organismal complexity decreases. As a result, the mathematical influence of the chessboard is likely to be more prominent for taxonomic groups with low (e.g. plankton) than great (e.g. mammals) biocomplexity. Our study therefore reveals the complex interplay between a fundamental mathematical constraint on biodiversity resulting from the interaction between the species’ ecological niche and fluctuations in the environmental regime (here, temperature), which has a predictable component and a stochastic-like biological influence (diversification rates, origination and clade age) that may alter or blur the former.

## Introduction

Understanding how life is arranged has occupied scientists for centuries [1–3]. Despite many hypotheses or theories [1, 3, 4] however, there is still no consensus on the causes of some marine ecogeographic patterns such as the Latitudinal Biodiversity Gradient (LBG). Our recent MacroEcological Theory on the Arrangement of Life (METAL) proposes that current patterns in marine biodiversity is driven by the interaction between the ecological niche of a species (*sensu* Hutchinson [5]) and the local environmental regime (mean and variability), which subsequently propagates at the community level [6–9].

The concept of the niche *sensu* Hutchinson [5] represents the combination of environmental tolerances and resources required by an organism. Hutchinson [5] conceptualised this notion with the so-called n-dimensional hypervolume, in which n ideally corresponds to all environmental factors. Using the ecological niche allows consideration of underlying processes (genetic and physiological) that are difficult to identify and parametrise for a large number of species. Interaction between the niche and climatic and environmental changes propagate from the species to the community and ecosystem levels, and are detectable from the smallest ecosystems to the whole ecosphere [6].

The METAL theory (hereafter referred to as METAL) offers a way to make testable ecological and biogeographical predictions to understand how life is organised and how it responds to global environmental changes, including climate change [6, 10]. At the organismal level, METAL explains (i) how species are distributed in space (species range) and time (phenology)[9], (ii) local changes in species’ abundance[10–13], (iii) species responses to climate change in time and space, including the inconsistency in the relationships between climate and species’ abundance[12], (iv) phenologic and (v) biogeographic shifts[9]. At the community level, METAL explains (i) some biogeographic laws or ecogeographic patterns (e.g. Rapoport’s pattern, infrequency law)[7], (ii) large-scale patterns in biodiversity[7], (iii) changes in biodiversity in space and time[8], (iv) long-term community shifts, including environmentally-induced abrupt community shifts (also called regime shift)[14] and (v) species and community sensitivity and vulnerability to climate change[8]. In addition, METAL unifies all these different biological and ecological responses to environmental changes in space and time by a unique process: the interaction between the species ecological niche and fluctuations in the environmental regime (S1 and S2 Text).

When we applied METAL at a global scale and community level, it provides an assessment of the ecogeographic patterns in biodiversity[7, 8]. Not all potential niches are fully occupied in real communities however, a phenomenon called niche vacancy or saturation[15]. Therefore, METAL can be seen as providing an estimation of the species richness at saturation. Because niche saturation is the ratio of observed species richness of a given taxonomic group on the modeled (from METAL) theoretical number of species at saturation, the procedure enables large-scale patterns in niche saturation to be assessed when applied in conjunction with data for observed species richness of different taxonomic groups[4]. Investigating the LBG and ecogeographic patterns in niche saturation jointly in this way may provide important clues to the key factors and processes that influence the arrangement of marine life.

Here, using METAL [6–9] and global-scale observations [4], we reconstruct global oceanic biodiversity and examine large-scale ecogeographic patterns in niche saturation for six taxonomic groups (foraminifers, euphausiids, oceanic sharks, tuna/billfish, cetaceans, pinnipeds). We investigate what factors or processes may be at the origin of those ecogeographic patterns to help identify the respective influence of ecological and evolutionary factors. The influence of diversification processes such as speciation and extinction is studied indirectly by the examination of niche saturation, which integrates (theoretical) expected richness at saturation and observed species richness of the six taxonomic groups considered in this study. We investigate whether the mean degree of niche saturation of a taxonomic group depends upon organismal complexity and discuss briefly, how climate change may restructure current patterns of marine biodiversity. Based on those findings, we propose a new hypothesis using the analogy of a chessboard that integrates both ecological and evolutionary processes in a single theoretical framework to explain the arrangement of life in the superficial (epipelagic) part of the ocean.

## Materials and methods

### Physical data

Annual SSTs originated from the dataset ERSST_V3 (1850–2014). This dataset is derived from a reanalysis based on the most recently available International Comprehensive Ocean-Atmosphere Data Set (ICOADS). Improved statistical methods have been applied to produce a stable monthly reconstruction, on a 1° x 1° spatial grid, based on sparse data[16]. Both mean annual SST and SST variability (i.e. the coefficient of variation of SSTs) were calculated on the basis of monthly SSTs for the period 1850–2014; a total of 165 years x 12 months = 1980 values were therefore used to assess mean annual SST and SST variability in each geographical cell. SST variability was subsequently scaled between 0 and 1 as follows:

$$g^{*}=\frac{g-\text{min}(g)}{\text{max}(g)-\text{min}(g)}$$ (1)

With g* the scaled variable and g SST variability. We used the coefficient of variation of SSTs instead of the standard deviation to remove the positive influence of the mean of SSTs on the standard deviation. We prefer this parameter to the range of SSTs, which may be affected too much by extreme values at a global scale. The coefficient of variation of SSTs have been frequently used in the past as an index of temporal variability of SSTs [17–19].

### Biological data

The dataset of observed biodiversity was provided by Dr Derek Tittensor, Dalhousie University [4]. The data were compiled from empirical sampling data (foraminifers and bony fish) or from expert-verified range maps (euphausiids, oceanic sharks and marine mammals) encompassing many decades of records. The data was originally gridded on a 880-km equal-area resolution grid [4]. The taxonomic groups we chose had a species richness that reflected well its biodiversity (i.e. species richness greater than 50% of known species; see their Table 1): foraminifers (Chromalveolata Rhizaria, 39 species; 88% of known species), euphausiids (Crustacea Malacostraca, 86 species; 100% of known species), oceanic sharks (Chondrichthyes, 27 species; 100% of known species), tuna/billfish (Osteichthyes, 12 species; 63% of known species), cetaceans (oceanic marine mammals, 81 species; 96% of known species) and pinnipeds (sea/land marine mammals, 36 species; 100% of known species); this last taxonomic group shows a well-known inversed latitudinal biodiversity pattern[4], which is explained when biodiversity is decomposed into groups with different levels of steno/eurythermy [7]. Spatial representations of these data are available in Tittensor and colleagues (their Fig 1) [4].

**Table 1:** Median values of niche saturation for 4 ecological guilds. The values are assessment based on the calculation of the median value for all geographical squares with and without correction to account for species remaining to be named. The first correction was based on Appeltans and colleagues[36] and the second, only applied for plankton, was based on the work of TARA [41]. See Fig 9.

Ecological guilds	Estimates (no correction)	Estimates (correction based on Appeltans et colleagues[36])	Estimates (correction based on TARA expedition[41])
**Protozooplankton**	14.4699	23.8157	74.6600
**Metazooplankton**	37.6993	42.9136	49.4985
**Fish**	7.6174	9.0936	-
**Marine mammals**	0.6674	0.7123	-

**Fig 1:**
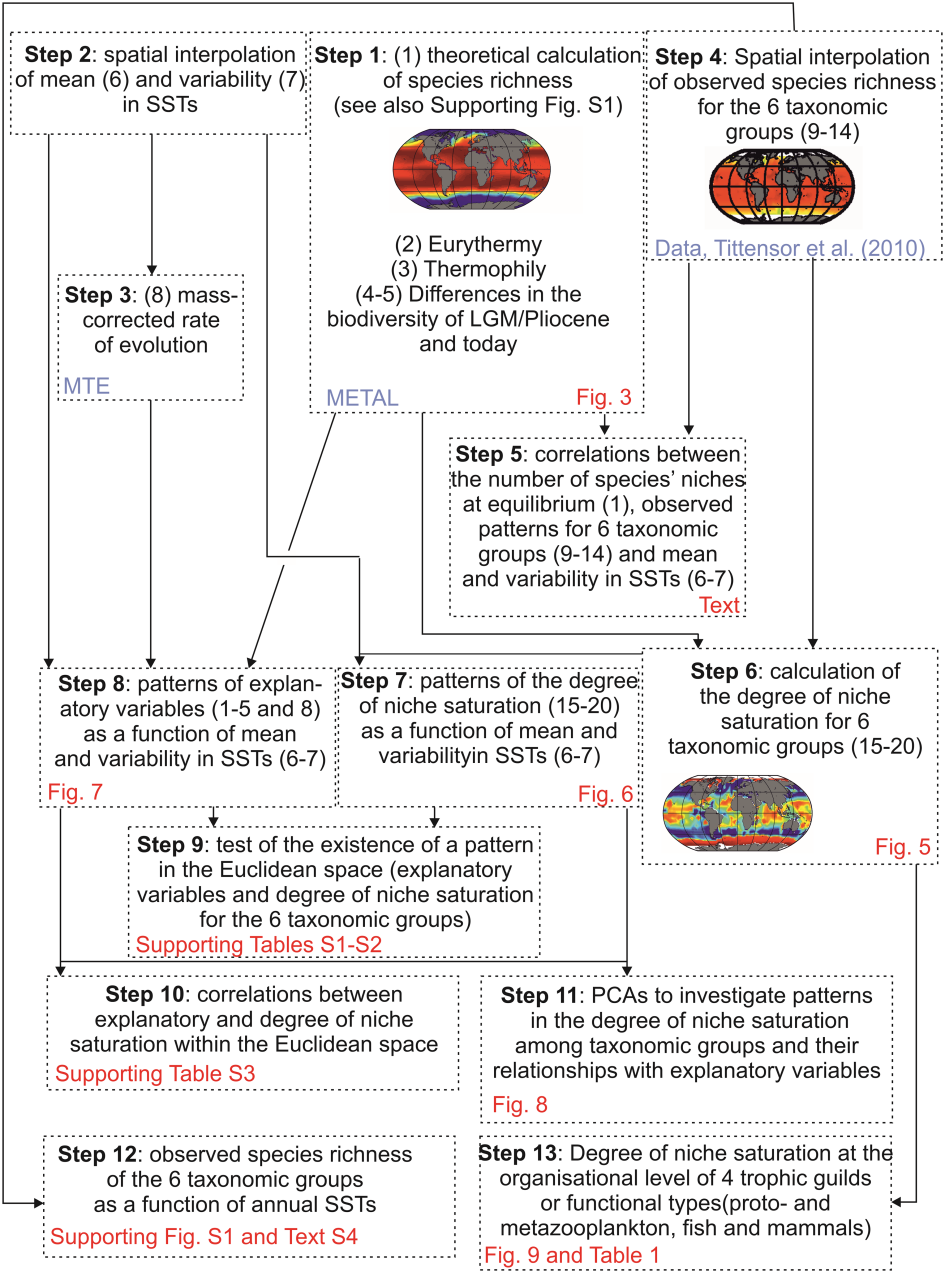
Sketch diagram that summarises the procedures and analyses used in this paper. MTE: use of the Metabolic Theory of Ecology. METAL: use of the MacroEcological Theory on the Arrangement of Life. LGM: Last Glacial Maximum. SST: Sea Surface Temperature. PCA: Principal Component Analysis.

### Analyses

The analyses were conducted in 13 steps (Fig 1).

#### Step 1: Estimation of theoretical patterns in pseudo-species richness and other key explanatory variables from the MacroEcological theory on the arrangement of life

The MacroEcological Theory on the Arrangement of Life (METAL): we first reconstructed large-scale biodiversity patterns using a model based on the MacroEcological Theory on the Arrangement of Life (METAL) [6, 8, 9, 14](S1 Text). This theory explains how life is arranged in the sea and how changing environmental conditions alter biological arrangements in space and time at different organisational levels (e.g. species, community, ecosystem), allowing precise predictions to be tested. In particular, we use the model recently developed in Beaugrand and colleagues[8] that estimates the implications of climate change on biodiversity for the past, present and future. This model is based on the use of thermal niches and temperature. While temperature is the most frequently used environmental parameter in theories or empirical studies we are nevertheless aware that the niche is a multi-dimensional concept[20] and that other environmental parameters affect species[2](S2 Text). While additional environmental parameters are probably necessary to provide better estimations on smaller spatial scales we assume that temperature alone provides a correct estimation of theoretical biodiversity patterns on a global scale; this assumption was tested in our previous studies that showed temperature explains a large proportion of variance of both past and contemporaneous patterns in biodiversity for several taxonomic groups[7, 8, 10]. More information on the METAL and its applications can be found in http://metaltheory.weebly.com/.

**Fig 2:**
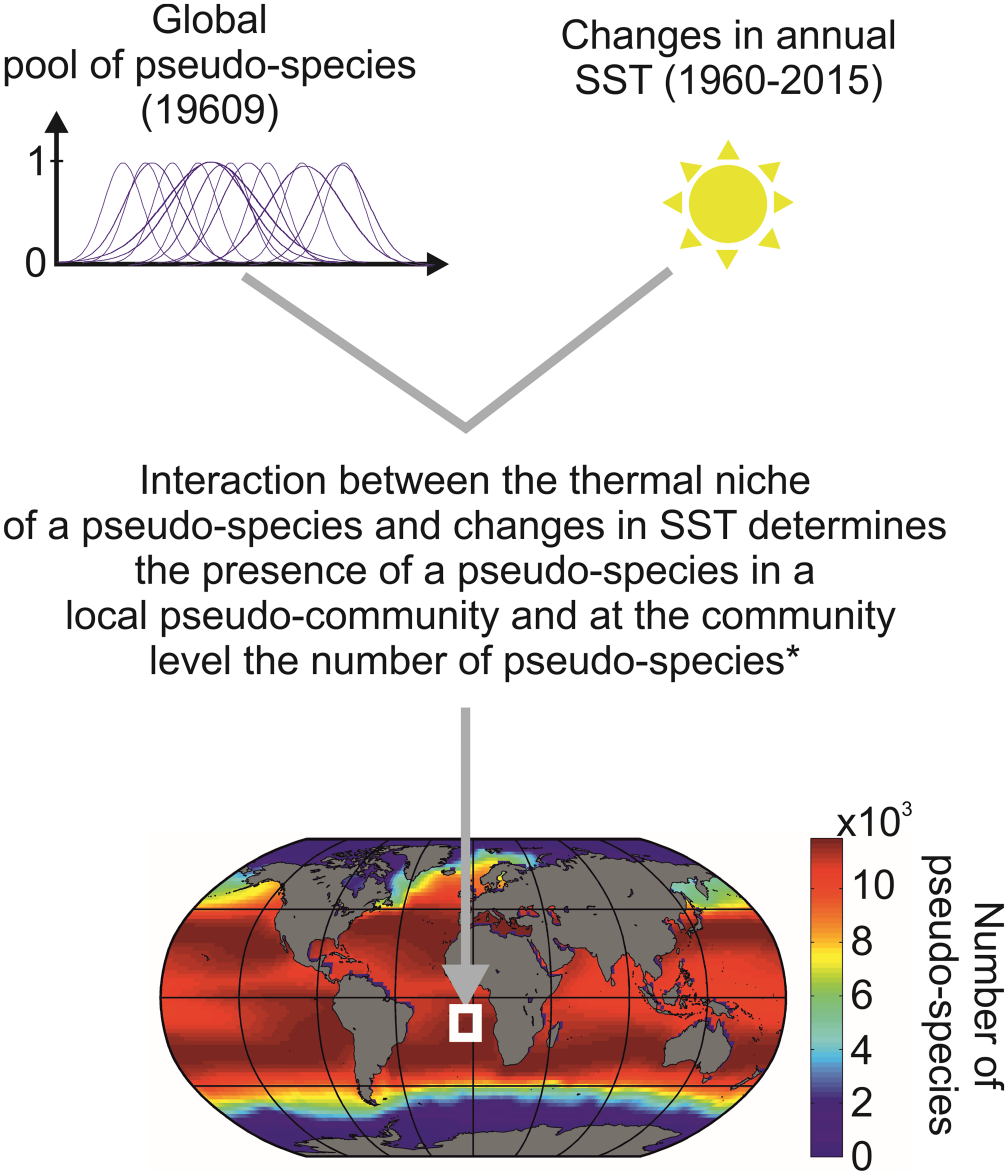
Schematics that illustrate how the METAL model generates pseudo-species and associated ecological niches to assess pseudo-species richness and the total number of niche at saturation in all oceanic areas. *: a pseudo-species is considered to be present in a geographical cell when its expected annual abundance (between 0 and 1) is > 0.1.

Calculation of potential niches at saturation (variable 1 in Fig 1): a fundamental assumption of METAL is that each species has a unique ecological niche[21], which determines to a large extent its spatial (biogeography) and temporal (phenology and year-to-year to long-term changes) distribution[6–10, 12]. Providing this assumption is true, we can create a pool of pseudo-species, characterized by all thermal optima from low to high and thermal breadth from strict stenotherms to universal eurytherms. Each species, whether ectothermic or endothermic, has evolved anatomical structures and life history traits that allow them to live within a certain range of thermal conditions. In other words, all species have their own thermal niche and so we expect the model will apply to both ectotherms and endotherms. All pseudo-species colonise a given region of the global ocean providing they can withstand the local thermal (annual SST) regime (Fig 2). By this way, we reconstruct the arrangement of life in the oceans and seas and the number of maximal species’ niches at saturation is approached by using the total number of pseudo-species per geographical square [7, 8].

More specifically, pseudo-species richness S^t^ was assessed by using the METAL model of Beaugrand and colleagues [8]. The response curve of the abundance L of a pseudo-species s in a given site i and time j to change in annual SSTs (1960–2013, S2 Text) was modeled by the following Gaussian function [22]:

$$L_{i,j,s}=c_{s}e-\left(\frac{{\left(\lambda_{i,j}-\phi_{s}\right)}^{2}}{2h_{s}^{2}}\right)$$ (2)

With L_i,j,s_ the expected abundance of a pseudo-species s at location i and time j (years of the period 1960–2013); c_s_ the maximum value of abundance for species s fixed to one; λ_i,j_ the value of temperature at location i and time j; ϕ_s_ the thermal optimum and h_s_ the thermal breadth for species s (this parameter corresponds to the standard deviation in the normal distribution). Once the niche was modeled, the expected abundance of pseudo-species was determined in space or time by linear interpolation from annual SST for a given year or time period and geographical square. A large number of pseudo-species was created with ϕ_s_ varying between -1.8°C and 40°C by 0.1°C increments and h_s_ varying between 1.1°C and 10°C by increments of 0.05°C. As we showed previously [7], this implementation generates a Mid-Domain Effect (MDE)[23] in the Euclidean space of the niche, which influences the geographical patterns in modeled species richness and niche saturation because more thermal niches overlap in warmer than in colder environments [7]. Our model assumes that no species of the same ecological guild can have exactly the same thermal niche, although relaxing this constraint would not alter our conclusions significantly. In contrast to our framework, some biodiversity models (empirical or geometrical) do not assume niche exclusion [24, 25].

**Fig 3:**
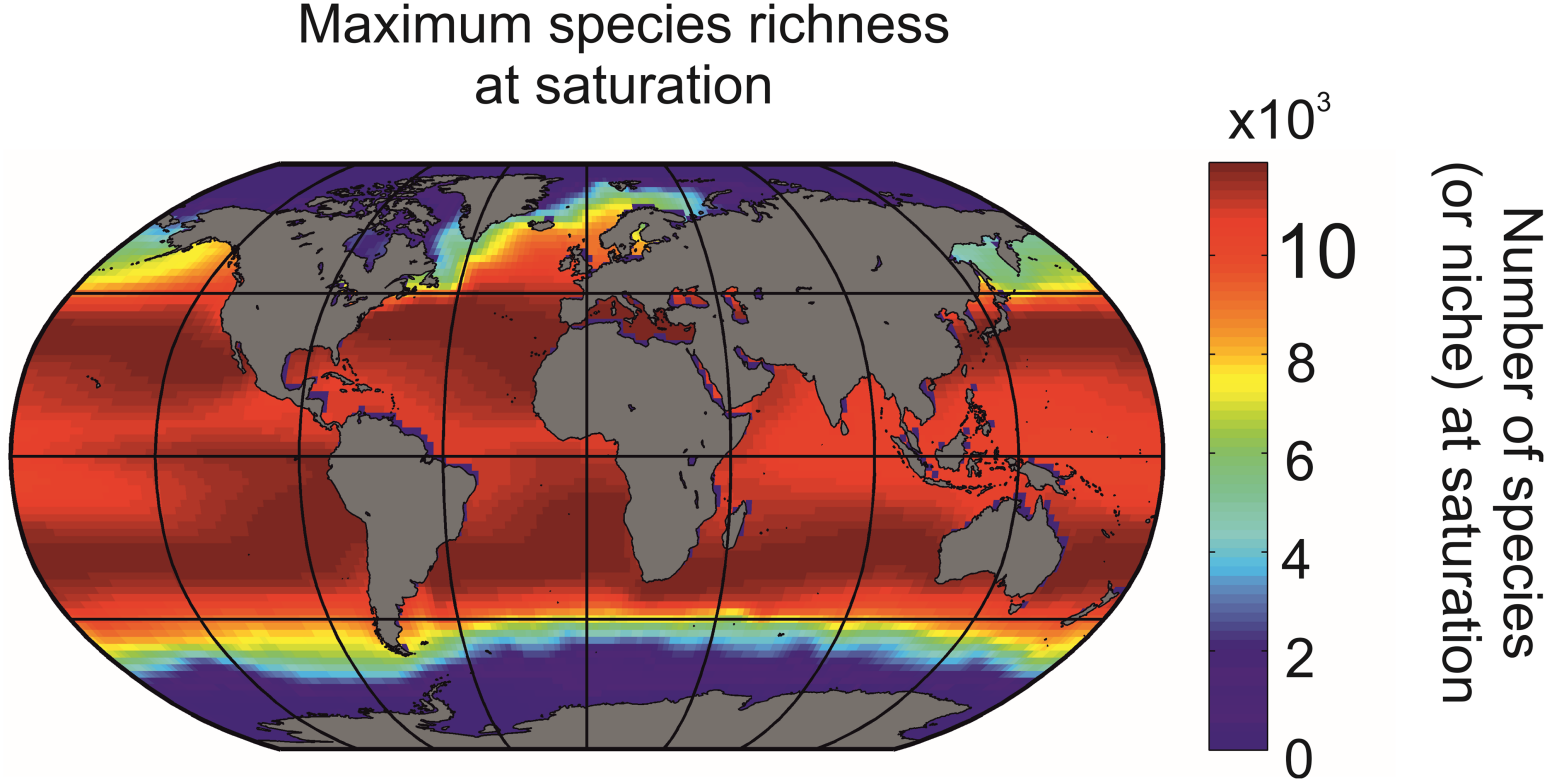
Biodiversity modeled by the METAL theory, indicating the (theoretical) maximum number of species’ niches at saturation.

After removing randomly half of the created pseudo-species to reduce calculation time [8], a total of 19609 pseudo-species was able to colonise the whole ocean [8]; we showed previously that this does not affect the ecogeographic biodiversity patterns (Beaugrand and colleagues [7], see Scenarios 18–19 and 27 in their S1 Table). The map of biodiversity, which can also be interpreted as a map of the maximal number of species’ niche at saturation is displayed in Fig 3. Data are available S1 Data.

Degree of eurythermy and thermophily (variables 2 and 3 in Fig 1): from the METAL model [8], we calculated the degree of eurythermy of a pseudo-community (i.e. weighted mean of the degree of eurythermy for all pseudo-species) and the degree of thermophily of each pseudo-community (i.e. weighted mean of the degree of thermophily for all pseudo-species) per geographic square. These two properties were calculated to better understand ecogeographic patterns in the degree of niche saturation of the six taxonomic groups. We represented the degree of eurythermy using 10 categories, each representing a decile; for example, the first and last categories corresponded to the first and last deciles.

Differences in biodiversity between the Last Glacial Maximum/mid-Pliocene and today (variables 4 and 5 in Fig 1): data on quantitative changes in biodiversity expected between the Last Glacial Maximum (LGM, ~20,000 years ago) and today, and between the mid-Pliocene (mid-Piacenzian, 3.264–3.025 Ma) and today, originated from the model of Beaugrand and colleagues [8]. The index of quantitative change was the sum of the differences between theoretical abundance (standardized between 0 and 1) at two time periods weighted by species richness common for the two time periods. This index was calculated for both periods for pseudo-species having an abundance higher than 0.1 (a threshold below which the species was considered to be absent) for both periods. For a given geographical cell, quantitative index F was calculated as follows:

$$F=\frac{1}{p}\sum_{i=1}^{p}\left|a_{i}-b_{i}\right|$$ (3)

With p species richness common to the two time periods, a_i_ the abundance of species i at the second time period and b_i_, the abundance of species i at the first time period. In this model, the maximum abundance of all species reaches 1. Those gridded data were used as a proxy for environmental perturbations induced by long-term climate change.

All biological variables assessed from METAL were calculated on a spatial grid of 2° longitude x2° latitude from 178°W to 180°E and from 88°S to 88°N.

#### Step 2: Spatial interpolation of mean and variability in SSTs (variables 6 and 7 in Fig 1)

Mean and variability in SSTs were spatially interpolated on a grid of 2° longitude x2° latitude from 178°W to 180°E and from 88°S to 88°N using the inverse squared distance method [26].

#### Step 3: Estimation of mass-corrected rate of evolution from mean SSTs (variable 8 in Fig 1)

Although the Metabolic Theory of Ecology (MTE) remains debated [27], we used a model originating from this framework to calculate the mass-corrected evolutionary rate [28]. The mass-corrected evolutionary rate (here the rate of DNA substitution; mitochondrial DNA) D was calculated from annual SST [29]:

$$D=-\frac{\alpha }{kV}+\mu$$ (4)

With α = -0.74eV, k the Boltzmann’s constant (8.62 x 10^−5^ eV.K^-1^), V the mean annual SST (K) and μ = 30.28 [29]. Values of α and μ originated from the linear model of Gillooly and colleagues based on mitochondrial DNA data [29]. Those data were utilized to examine the relationships between niche saturation and a proxy for evolutionary rates.

#### Step 4: Spatial interpolation of observed species richness (variables 9–14 in Fig 1)

Observed species richness for foraminifers, euphausiids, tuna/billfish, oceanic shark, cetaceans and Pinnipeds were also spatially interpolated on a grid of 2° longitude x2° latitude from 178°W to 180°E and from 88°S to 88°N using the inverse squared distance method [26].

#### Step 5: Correlation between expected and observed large-scale biodiversity patterns

Linear correlations between expected and observed biodiversity patterns were calculated for each taxonomic group and between expected biodiversity and both mean and variability in SSTs (Fig 1). To account for spatial autocorrelation in the geographical pattern of species richness (two dimensions), the degrees of freedom were recalculated to indicate the minimum number of samples (n*) needed to maintain a significant relationship at p = 0.05 [19, 30, 31]. The smaller n*, the less likely is the effect of spatial autocorrelation on the probability of significance [7]. We preferred this technique to others (e.g. a technique based on the calculation of the Moran's index or classical semi-variograms) that are based on the assumption of isotropy, which is often violated, as shown on the diversity of North Atlantic calanoid copepods calculated by using (local) Point Cumulative Semi-Variograms [32].

#### Step 6: Degree of niche saturation

**Fig 4:**
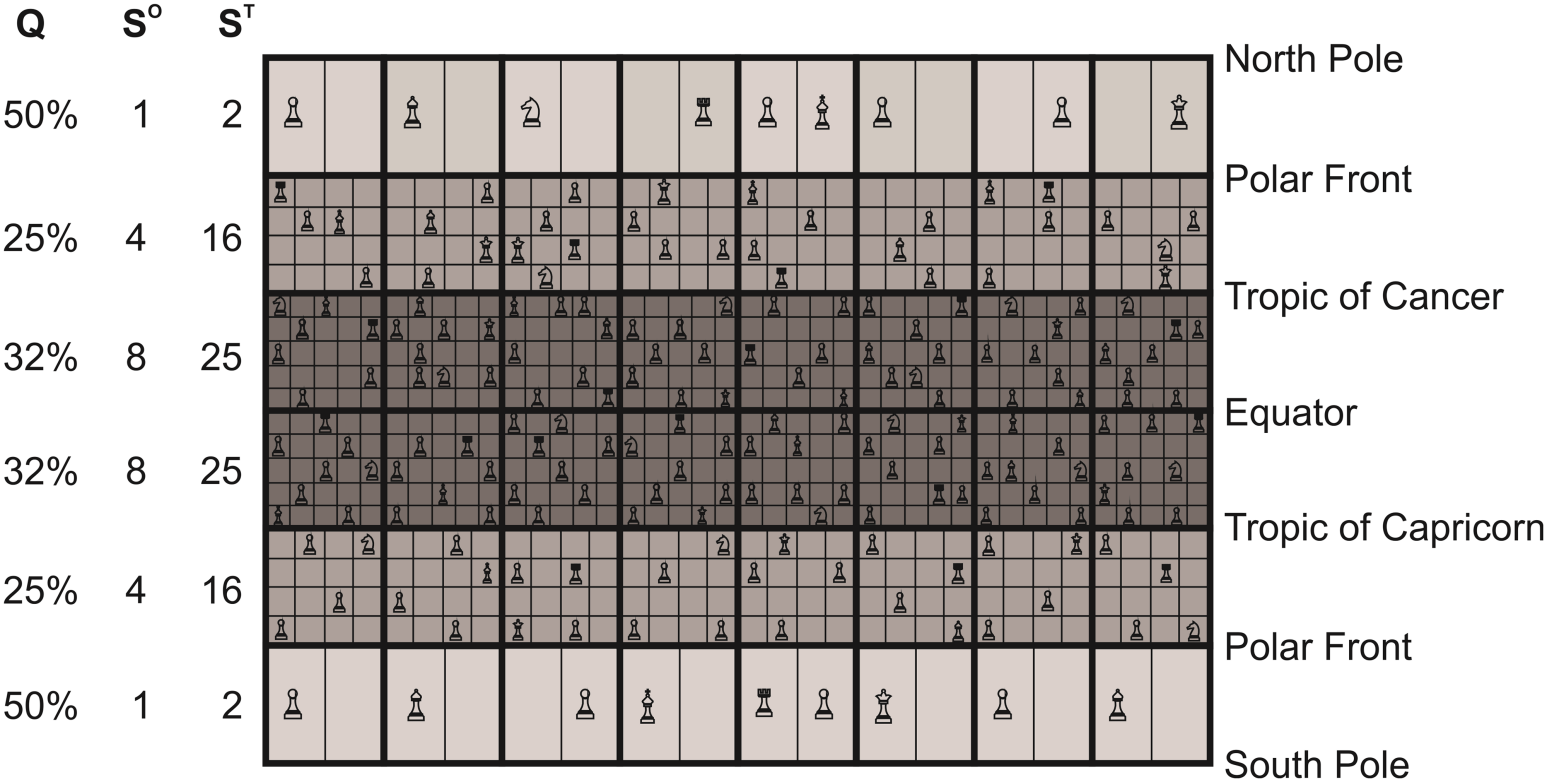
Schematics that illustrate our hypothesis on how the life chessboard influences ecogeographic pattern in marine biodiversity. This peculiar chessboard has a number of squares that corresponds to the number of geographic squares that divides the oceans. In each square, a sub-square represents a specific ecological niche *sensu* Hutchinson [20] and their number in a square illustrates maximum species’ niches at saturation. Note that the area of a sub-square should not be not interpreted as related to the size of a niche. Future research may show that it is related to the number of individuals within a species, although this also depends upon resource availability and environmental conditions. Sub- squares are not referenced in the geographic space and represent a potential niche that can be taken by a species (not an individual). In this study, the number of sub-squares is fixed by the MacroEcological Theory on the Arrangement of Life (METAL). Q means niche saturation of a given community. S^0^ and S^T^ represent observed species richness and maximum species’ niches at saturation (expected from METAL), respectively. The species, the chess piece on the chessboard, can appear and disappear by speciation (and immigration) and (local or global) extinction, respectively. The different chess pieces illustrate the differences in species’ life history traits such as dispersal (e.g. rooks and bishops move in a different way) and degree of importance or trophic status (e.g. a queen has not the same importance than a pawn). Although marine biodiversity should increase from poles to the tropics, clade origination and life history traits of a specific taxonomic group may blur this pattern. The increase in diversification rates from cold to warm regions or the increased probability of clade origination in the tropics where the number of niches are greater explain an augmentation in niche saturation between temperate and permanently stratified regions because the temperate-tropic difference in maximum species’ niches at saturation is small nowadays. Biogeographic shifts can rearrange the sub-squares in a geographic square and when occupied the chess pieces (species), providing that there is no geographical barrier or that dispersal is sufficiently elevated. In case of a global cooling, the Latitudinal Biodiversity Gradient (LBG) reinforces because of the movement of the sub-squares and associated species towards the equator. In case of global warming, the LBG is attenuated because of the movement of sub- squares and corresponding chess pieces polewards.

The degree of niche saturation can be defined as the percentage of occupied niches (*sensu* Hutchinson [5]) in a particular community (see Fig 4 for an example). For a given taxonomic group or an ecological guild, niche saturation can be assessed by calculating the number of species observed in a given location out of the maximum number of species that can coexist in a site [15]; this way of calculating saturation is similar to what was proposed by MacArthur and Wilson in their equilibrium theory of insular zoogeography [33] (page 373). For each of the six taxonomic groups, the degree of niche saturation Q_x_ of a taxonomic group x for a given geographical cell was determined by calculating the ratio of the observed species richness $S_{x}^{o}$ on the maximum species’ niche at saturation $S_{y}^{t}$(theoretical biodiversity assessed from METAL in each geographical cell and seen in Fig 3)

$$Q_{x}=\frac{S_{x}^{o}}{S_{y}^{t}}\text{for}\;S_{y}^{t}\geq 1$$ (5)

**Fig 5:**
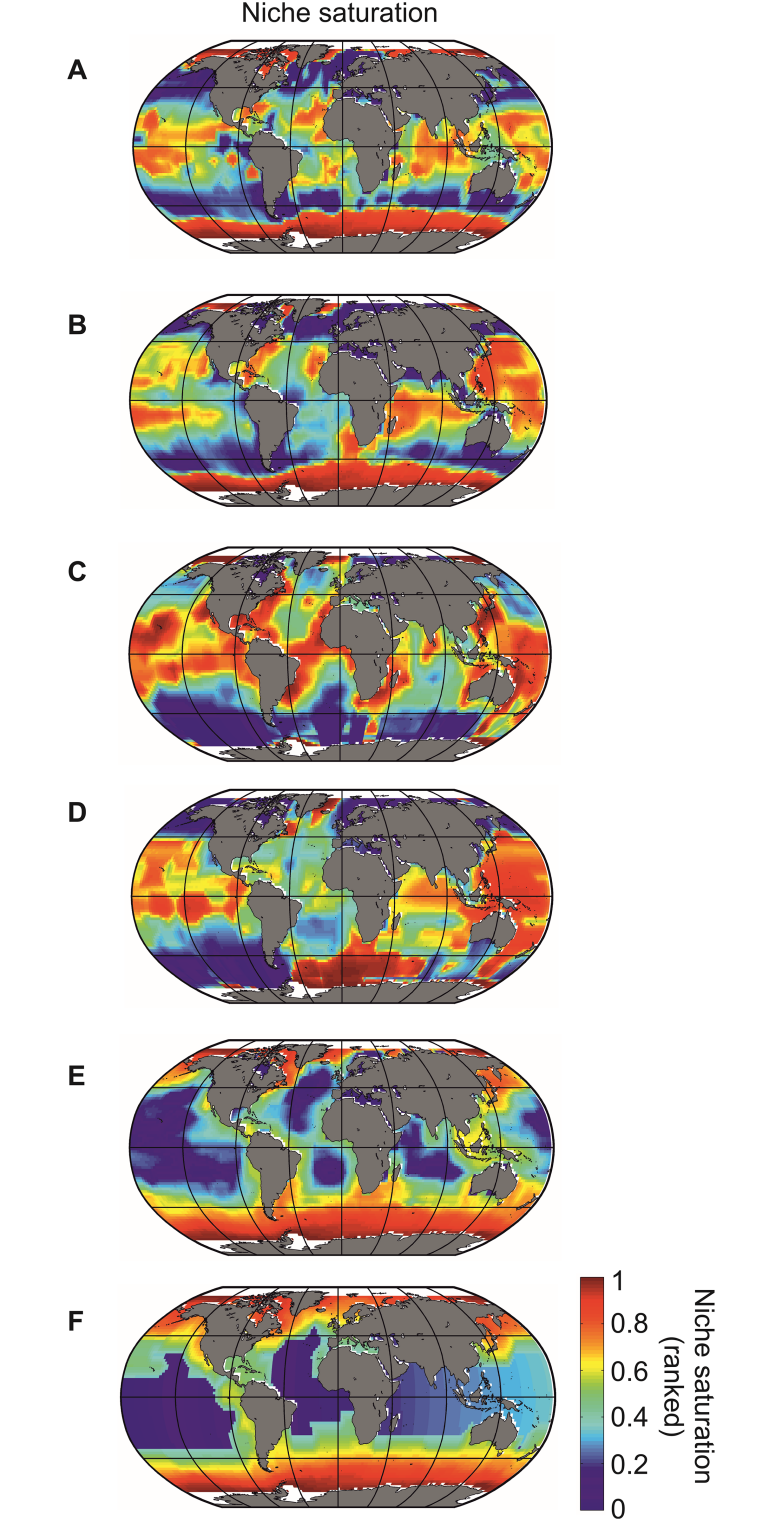
Niche saturation for (A) foraminifers, (B) euphausiids, (C) oceanic sharks, (D) tuna/billfish, (E) cetaceans and (F) pinnipeds. For niche saturation, when values tend to 1, this indicates a large degree of niche saturation, and inversely. Oceanic areas in white are missing data either because of the proximity of a geographical square to the coast or high sea ice concentration.

The total number of niches (or species) at saturation considered in our model was 19609. The oceans contain between ~200000 and ~250000 already inventoried species [34, 35], which represents between 7.8% and 9.8% of all described species and between 0.9 and 3.9% of all estimated species (between 0.5 and 2.2 million species [35, 36]). The taxonomic groups we considered represented between 0.06% (12 species of tuna/billfish) and 0.44% (86 species of euphausiids) of the modeled biodiversity (19609). Results from the calculation of Q for the 6 taxonomic groups (foraminifers, euphausiids, oceanic sharks, tuna/billfish, cetaceans and pinnipeds) are shown in Fig 5. To ensure that maps of niche saturation were comparable, we replaced niche saturation values by rank and scaled them between 0 (complete unsaturation) and 1 (full saturation). From Eq (5), we see that $S_{y}^{t}$ did not vary among taxonomic groups, only among geographical cells. In this analysis, we therefore examined ecogeographic patterns in relative saturation among groups and not directly their values of saturation (see Step 13 for the comparison of the values of niche saturation).

#### Step 7: Examination of the ecogeographic patterns in the degree of niche saturation of the six taxonomic groups (variables 15–20 in Fig 1) according to annual SSTs and SST variability (variables 6–7 in Fig 1)

**Fig 6:**
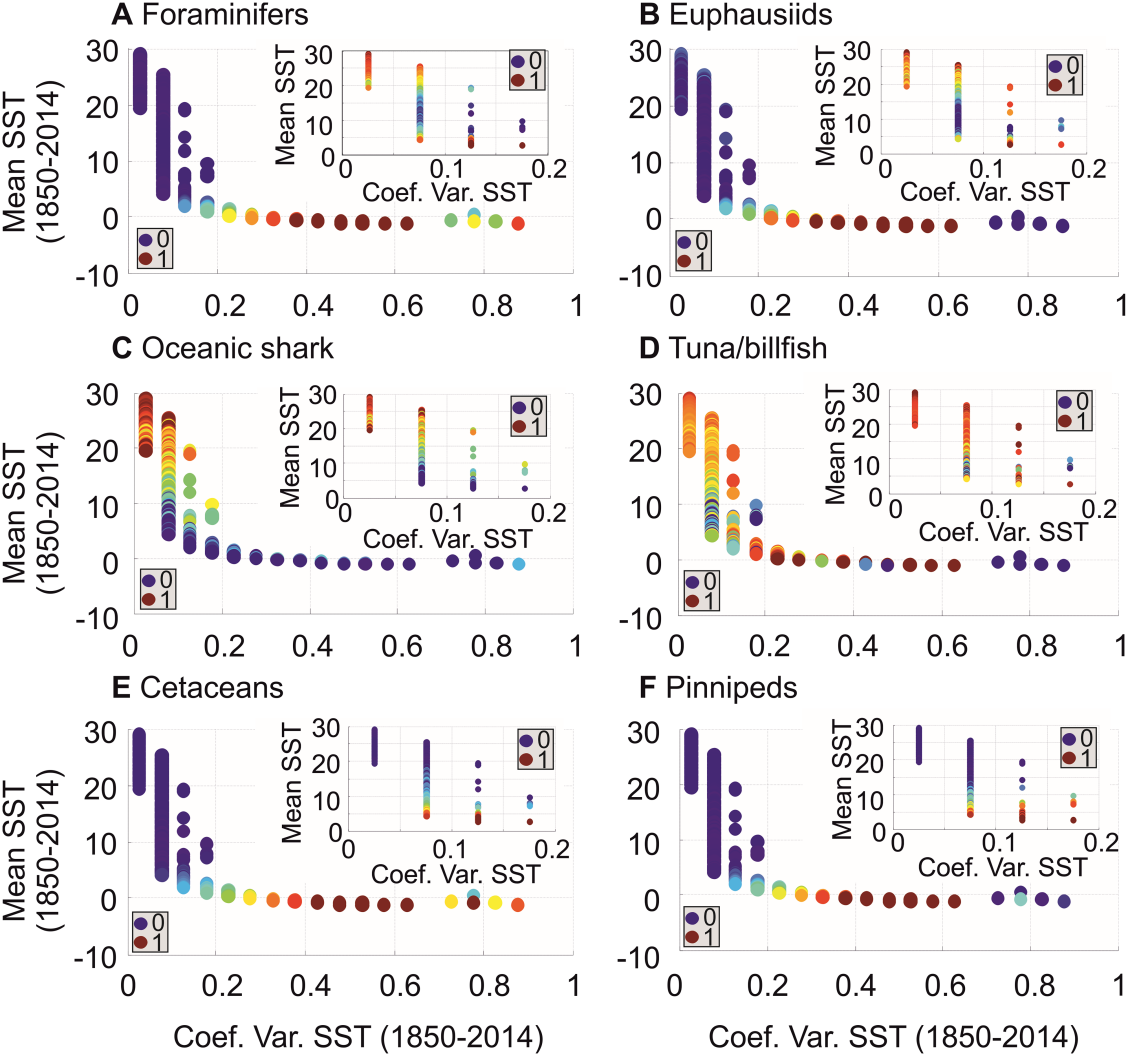
Degree of niche saturation for the pelagic ocean of six taxonomic groups as a function of mean SST (between -1°C and 30°C) and SST variability. **(A)** foraminifers, **(B)** euphausiids, **(C)** oceanic sharks, **(D)** tuna/billfish, **(E)** cetaceans and **(F)** pinnipeds. The inner Figure for each taxonomic group is a focus of niche saturation for mean SST ranging from 2.75°C to 30°C. All values of niche saturation were represented by bullets. Those values were standardized between 0 (blue bullet; lowest value) and 1 (red bullet; highest value). Lowest and highest values are indicated in each panel.

The degree of niche saturation of the six taxonomic groups (Fig 5) was assessed as a function of mean SSTs and SST variability (i.e. the coefficient of variation of SSTs). For this analysis, we used the matrices constructed to map the niche saturation of the six taxonomic groups in Fig 5 (Step 6) and the corresponding matrices of mean and variability in SSTs interpolated in Step 2 (Fig 1). A total of 8 matrices were therefore analysed and each corresponded to a grid of 2° longitude (from 178°W to 180°E) x 2° latitude (from 88°S to 88°N). The median of the degree of niche saturation was calculated for each interval of mean annual SST between -1°C and 30°C by increment of 0.25°C and each interval of SST variability between 0 and 1 by increment of 0.05; values of mean annual SST below -1°C, not shown, were under-represented and most were highly saturated so that they appear as outliers in our subsequent analyses (principal component analyses). The median was only calculated when the minimum number of values of niche saturation inside a given range of mean and variability in SSTs was higher than 10. To minimize the influence of outliers on the graphics, we calculated the 5^th^ and 95^th^ percentiles and fixed values below or above to these thresholds. Data were then standardized between 0 (lowest value of niche saturation) and 1 (highest value of niche saturation). The last two transformations were not applied when data were analysed (e.g. polynomial regression, correlation and principal component analyses; see below). The results of this analysis were represented on a scatterplot for each taxonomic group, with bullets that had a colour and a size proportional to their value (Fig 6); small blue bullets indicated small median values of niche saturation for a given increment of mean and variability in SSTs and conversely, for large red bullets. Data are available in S1 Data.

#### Step 8: Examination of patterns in explanatory variables (variables 1–5 and 8 in Fig 1) according to annual SSTs and SST variability (variables 6–7 in Fig 1)

**Fig 7:**
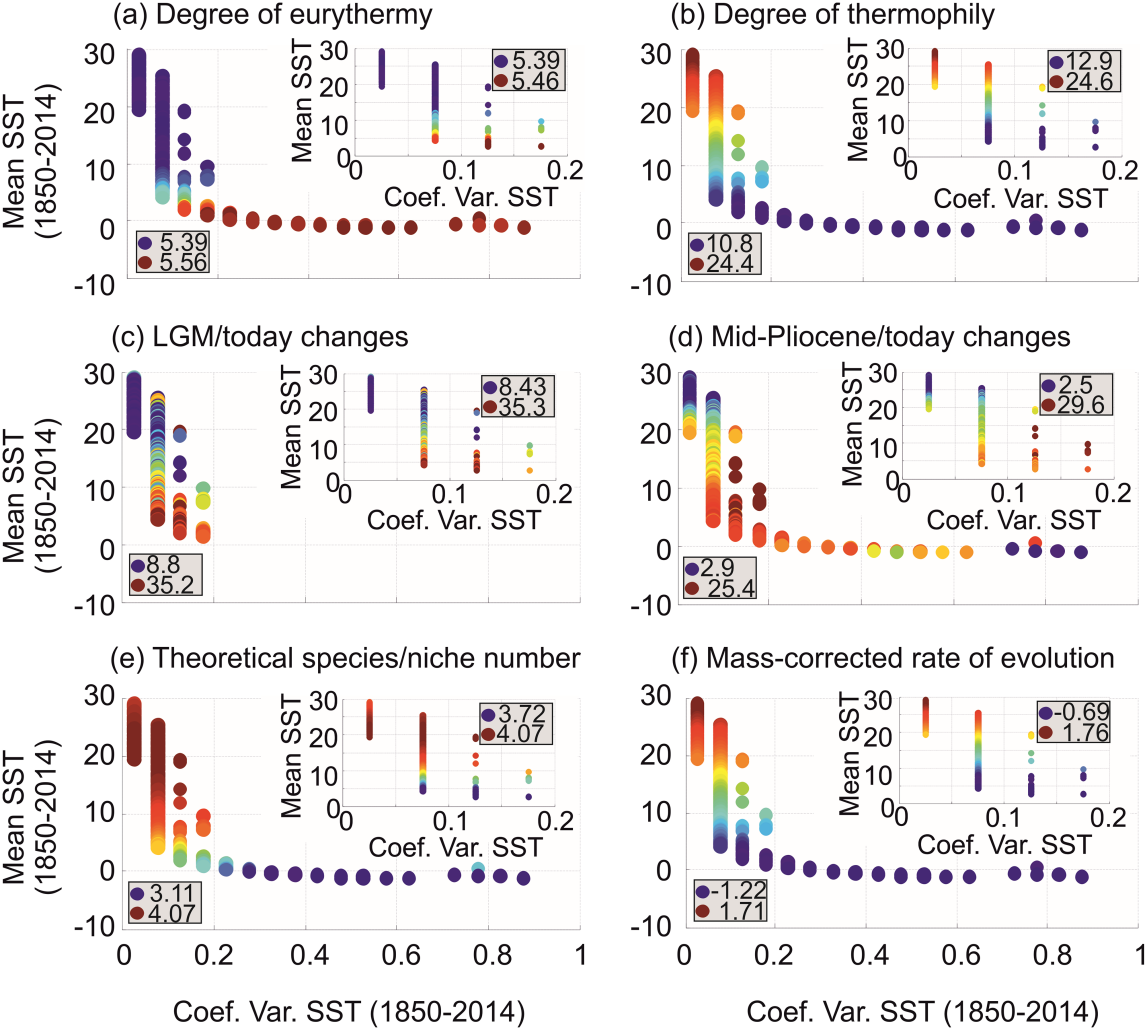
Explanatory variables as a function of mean SST (between -1°C and 30°C) and SST variability. **(A)** degree of eurythermy of the pseudo-community, **(B)** degree of thermophily of the pseudo-community, **(C)** quantitative changes in modeled (METAL) biodiversity between the LGM and today, **(D)** quantitative changes in modeled (METAL) biodiversity between the mid-Pliocene and today, **(E)** expected (METAL) theoretical number of niche/species at saturation and **(F)** mass-corrected evolutionary rate assessed from the MTE. Blue and red bullets denote low and high values in each explanatory variable, respectively. The inner figures for each explanatory variable are a focus of the variability in niche saturation for mean SST ranging from 2.75°C to 30°C. Values of each explanatory variable were represented by colored bullets with low values being indicated by small blue bullets and high values by red ones; lowest and highest values are indicated in each panel. The values of the inner panels can be outside the range of the values of the larger panel because of the standardization procedure.

A similar procedure was applied for explanatory variables for a mean SST between -1°C and 30°C (Fig 7): (1) degree of eurythermy of each modeled (METAL) pseudo-community, (2) degree of thermophily of each modeled (METAL) pseudo-community, magnitude of changes in modeled (METAL) pseudo-species richness between (3) the LGM or (4) the mid-Pliocene and today, (5) the maximal expected (METAL) number of niches (or species) at saturation and (6) the mass-corrected evolutionary rate assessed from the MTE. Note that the degree of eurythermy and thermophily was not assessed from observed data because we have only information on observed species richness and not individual species range (see Step 12).

Standardization between 0 and 1 was not needed. For this analysis, we used the matrices interpolated in Steps 1–3 (Fig 1). Therefore a total of 8 matrices (the 6 explanatory variables above and mean and variability in SSTs) was analysed, each having a size corresponding to a grid of 2° longitude (from 178°W to 180°E) x 2° latitude (from 88°S to 88°N). As in step 7, results of the procedure were represented for each explanatory variable by means of a scatterplot (Fig 7). Bullets had a colour and a size proportional to the value of the variable; small blue bullets indicated small median values of a given explanatory variable for a given increment of mean and variability in SSTs and conversely for large red bullets (Fig 7).

#### Step 9: Examination of the patterns in niche saturation within the 2-dimensional Euclidean space determined by mean SST and SST variability

A 2-order polynomial regression was calculated to test whether patterns in niche saturation were detected within the 2-dimensional Euclidean space determined by mean SST and SST variability. R^2^, adjusted R^2^ and RMSE (Root Mean Squared Error) were examined. The basic polynomial model used for all regression analyses was:

$$\beta =\delta_{1}m+\delta_{2}g+\delta_{3}gm+\delta_{4}m^{2}+\delta_{5}gm^{2}+ϒ$$ (6)

With β the estimation of the degree of niche saturation of a taxonomic group (foraminifers, euphausiids, oceanic sharks, tuna/billfish, cetaceans, pinnipeds) or the value of an explanatory variable (degree of eurythermy, degree of thermophily, LGM/today quantitative changes, mid-Pliocene/today quantitative changes, theoretical number of niches, mass-corrected rate of evolution), m mean SST and g SST variability. The parameters δ_1_ to δ_5_ and ϒ were constants assessed from the regression analysis. A total of 12 polynomial regression analyses was performed, one for each variable considered in Figs 6 and 7 (S1 and S2 Tables).

#### Step 10: Correlations between changes in niche saturation and explanatory variables according to mean SST and SST variability

We calculated linear correlation coefficients between changes in niche saturation for the six taxonomic groups and explanatory variables in Figs 6 and 7 (S3 Table). Those correlations were based on (i) mean SSTs ranging from -1°C to 30°C (185 degrees of freedom for all variables but LGM (157)) and (ii) mean SSTs from 2.75°C to 30°C (149 degrees of freedom for all variables).

#### Step 11: Principal Components Analyses (PCAs)

**Fig 8:**
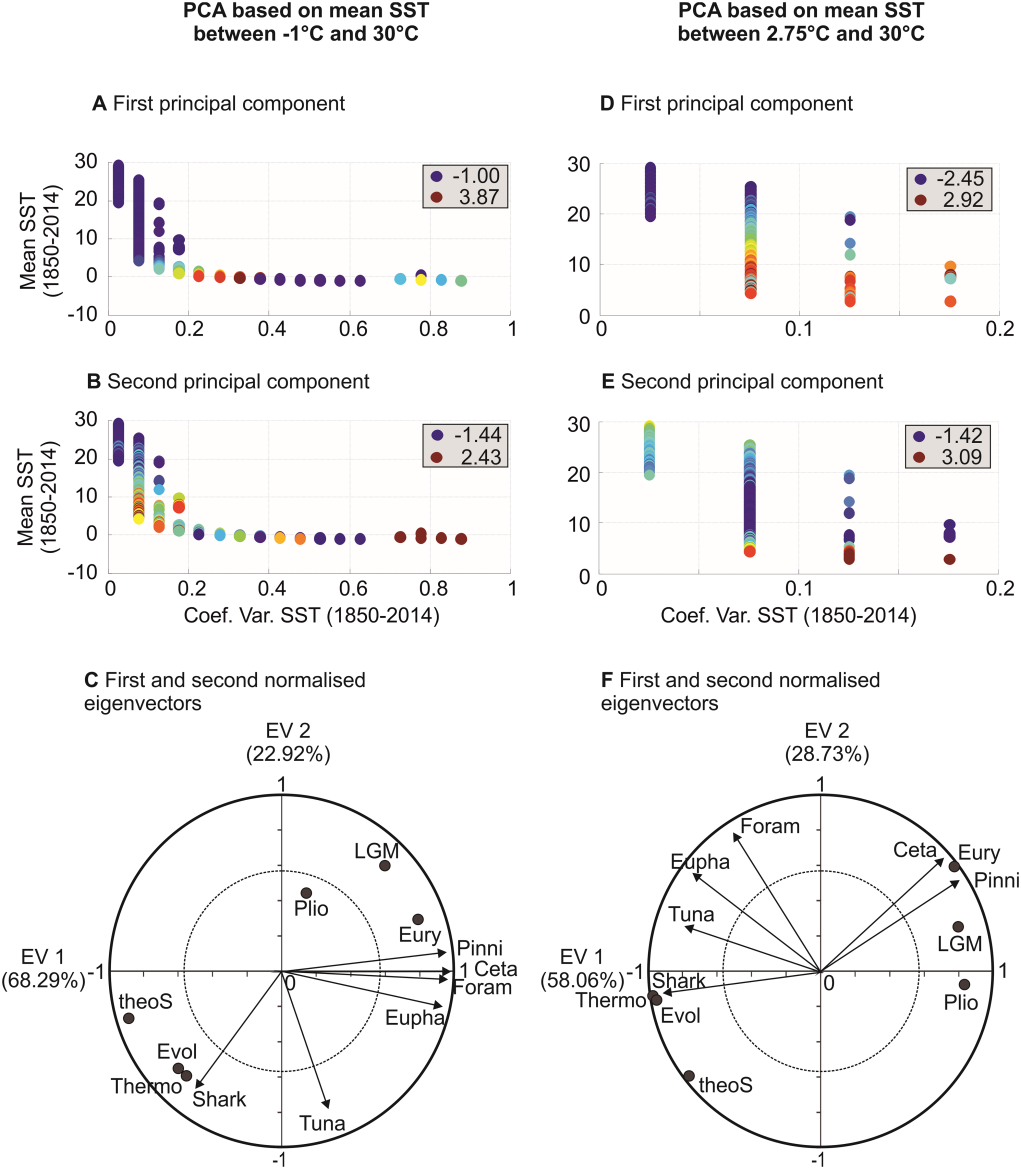
Principal Components Analyses (PCAs) on the table [degree of niche saturation as a function of mean SST and SST variability] x [6 taxonomic groups]. First PCA based on mean SST ranging from -1°C to 30°C. (**A**) First principal component (PC). (**B**) Second PC. (**C**) First and second normalized eigenvectors. Values of principal components were represented by colored bullets with low values being indicated by small blue bullets and high values by red ones; lowest and highest values are indicated in each panel. The dashed circle indicates the circle of equilibrium contribution. Second PCA based on mean SST ranging from 2.75°C to 30°C. (**D**) First PC. (**E**) Second PC. (**F**) First and second normalized eigenvectors. All variables outside this circle are well represented. Foram: foraminifers, Eupha: euphausiids, Shark: oceanic sharks, Tuna: tuna/billfish, Ceta: cetaceans, Pinni: pinnipeds. Supplementary variables (black bullets) are pseudo-community eurythermy (Eury; Fig 3), pseudo-community thermophily (Thermo), LGM/today biodiversity changes (LGM), mid-Pliocene/today biodiversity changes (Plio), theoretical number of niche/species at saturation (theoS) and mass-corrected evolutionary rate (Evol).

Principal Components Analyses (PCAs) were performed to investigate globally the relationships between the degree of niche saturation of the six taxonomic groups and their relationships with explanatory variables (Fig 8). The first PCA (Fig 8A–8C) was applied on the table [category of mean SST and SST variability] x 6 variables, using all categories of mean SST (i.e. mean SST between -1°C and 30°C by increment of 0.25°C) and SST variability (i.e. SST variability between 0 to 1 by increment of 0.05)(see Figs 6 and 7). The second PCA (Fig 8D–8F) was applied on the table [category of mean SST and SST variability] x 6 variables, for categories of mean SST higher than 2.75°C. The second PCA was performed to examine more closely the relationships between niche saturation of the six taxonomic groups and explanatory variables once some outliers were removed. Eigenvectors were normalised as follows [37]:

$$U^{*}=U\Lambda^{\frac{1}{2}}$$ (7)

With U* being the matrix of normalised eigenvectors, U the matrix of eigenvectors and Λ the diagonal matrix of eigenvalues. Thus, the variables in the space of eigenvectors represented the linear correlation with the first and the second principal components [38]. We used this mathematical property to add supplementary variables (all explanatory variables), simply by calculating the linear correlation (Pearson correlation coefficient) between these variables and the first two principal components. Thus, explanatory variables had no weight in the calculation of the principal components. The first two normalized eigenvectors and corresponding principal components were represented (Fig 8).

#### Step 12: Observed species richness as a function of temperature

A bar diagram was performed for each of the 6 taxonomic groups (foraminifers, euphausiids, oceanic sharks, tuna/billfish, cetaceans and pinnipeds), to examine the mean observed species richness per annual SST, between -2°C and 30°C and by 0.5°C increment (S1 Fig and S4 Text). This analysis was performed to estimate the mean thermal optimum and thermal range of each taxonomic group. The highest values in a bar diagram indicated mean thermal optimum T_o_, which was assessed by calculating the average of the first 5 highest values of species richness in the diagram. The degree of equity in a bar diagram was used as an indication of the thermal range E_o_ of a taxonomic group; here this was assessed by calculating the coefficient of variation of observed species richness.

#### Step 13: Global average degree of niche saturation for four ecological guilds

**Fig 9:**
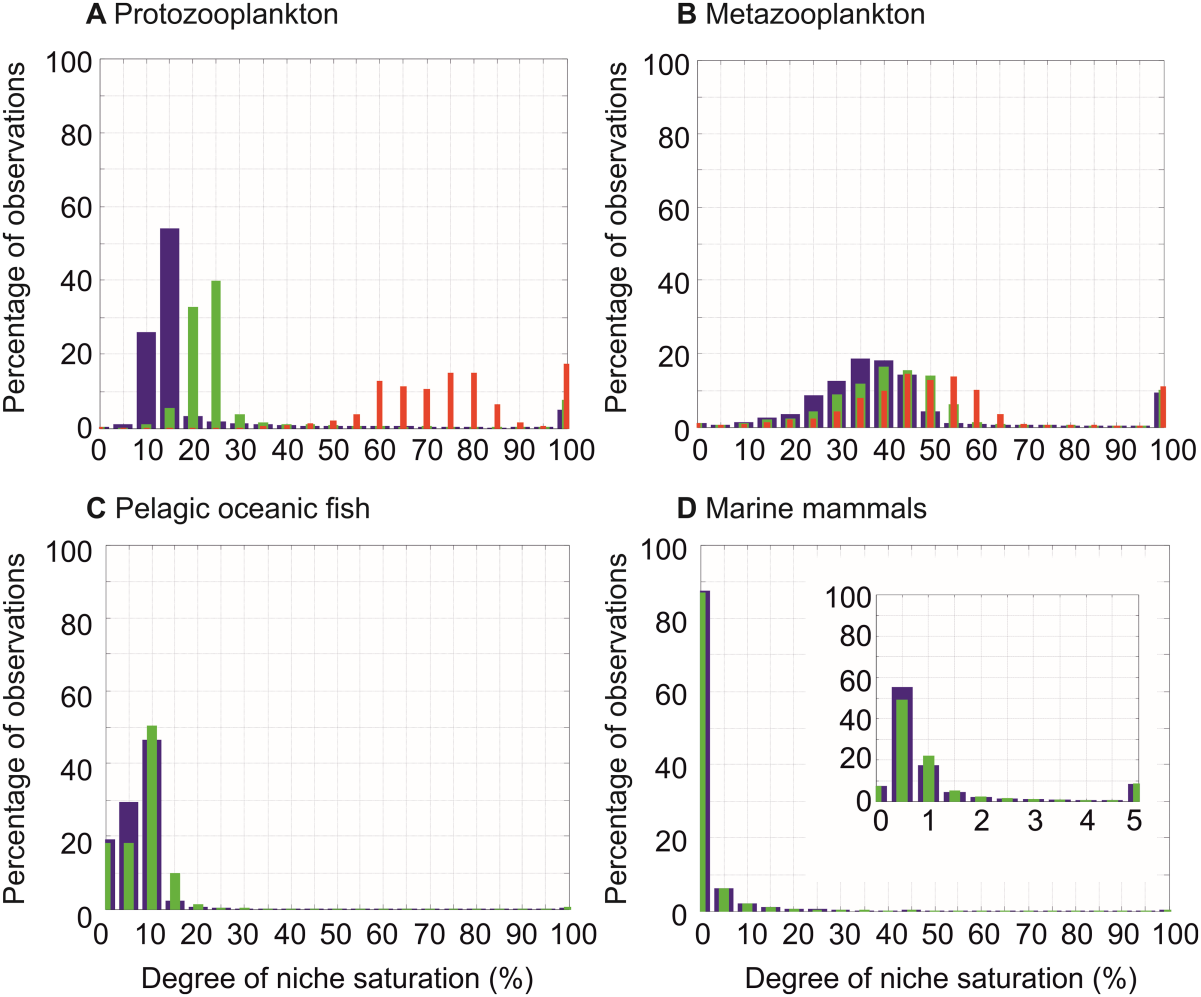
Histograms of niche saturation of four functional types in the oceanic hydrosphere. **(A)** Protozooplankton, **(B)** Metazooplankton, **(C)** pelagic oceanic fish and **(D)** marine mammals. The inner panel on (**D**) is a zoom of niche saturation between 0 and 5%. Blue bars exhibit values of niche saturation with no correction to account for species remaining to be described. Green bars are values of niche saturation corrected by using estimates based on Appeltans and colleagues[36] and red bars (only for plankton) are values of niche saturation corrected using estimates from TARA[41].

First estimations based on described species: we used the taxonomic groups as representative of some ecological guilds (or functional types) to assess their mean niche saturation as an absolute value (and not relative as in Fig 4); foraminifers (39 species) were assumed to be representative of protozooplankton, euphausiids (86 species) of metazooplankton, both tuna/billfish (12 species) and oceanic sharks (27 species) of pelagic oceanic fish and both cetaceans (81 species) and pinnipeds (36 species) of marine mammals (Fig 9).

Let $S_{x}^{o}$ be the species richness of a given geographical cell for a given taxonomic group x and $S_{y}^{t}$ the maximum (theoretical) species richness (or total number of niche at saturation) of a given geographical cell determined by the METAL model (o for observed species richness and t for theoretical pseudo-species richness). $S_{w}^{o}$ can be scaled for a given functional group or ecological guild w by dividing the total species richness of a taxonomic group x and by multiplying by the total species richness of the functional group or ecological guild w, as follows:

$$S_{w}^{o}=\frac{S_{x}^{o}}{x}w$$ (8)

x is the species richness of a given taxonomic group; x = 39 species for foraminifers, x = 86 species for euphausiids, x = 12 species for tuna/billfish, x = 27 species for oceanic sharks, x = 81 species for cetaceans and x = 36 species for pinnipeds. The parameter w was first fixed to 1350 for protozooplankton and 5500 for metazooplankton [39], 1068 for pelagic species [40] and 117 for marine mammals [4]. And similarly, $S_{y}^{t}$ can be scaled to correspond to the total species richness z of the whole species richness of all functional groups considered in the analysis. $S_{y}^{t}$ was divided by y (here 19609) and multiplied by z:

$$S_{z}^{t}=\frac{S_{y}^{t}}{y}z$$ (9)

z was first equal to 1350 (protozooplankton) + 5500 (metazooplankton) + 1068 (pelagic fish) + 117 (marine mammals) = 8035 species. The corrected degree of niche saturation Q_w_ of a trophic guild or functional type w is given by extrapolating species richness to a functional group and scaling maximum species richness to the total number of species considered in the analysis:

$$Q_{w}=\frac{S_{w}^{o}}{S_{z}^{t}}=\frac{\frac{S_{x}^{o}}{x}w}{\frac{S_{y}^{t}}{y}z}=\frac{S_{x}^{o}}{S_{y}^{t}}\frac{wy}{xz}=Q_{x}\frac{y}{x}\frac{w}{z}$$ (10)

With Q_x_ the degree of niche saturation of a given taxonomic group x calculated from Eq (5).

Uncertainties related to undescribed and unknown species: the estimations above are highly dependent upon the true species richness of an ecological guild. If estimations of global mean niche saturation of a given ecological guild are based on an underestimated number of species, this will lead to an underestimation in niche saturation. The conclusion could be that communities are highly undersaturated whereas they are not. Therefore, we also corrected our first estimations (see previous section) in different ways, depending upon the ecological guild (S3 Text).

For all ecological guilds, we corrected our estimations on mean niche saturation by adding species remaining to be discovered (S3 Text). Two corrections were applied for plankton, one based on the work of Appeltans and colleagues [36] and the other based on the work carried out as part of TARA expedition [41]. For protozooplankton, w (i.e. the total number of species in the ecological guild or functional type; here protozooplankton) was fixed to 2363 (first estimation[36]) and 30192 species (second estimation[41]) and z (i.e. the total number of species in all ecological guilds or functional types) to 9048 (first estimation[36]) and 36877 species (second estimation[41]) in Eqs (8)–(10). For metazooplankton, w was fixed to 8945 (first estimation[36]) and 22500 (second estimation[41]) species and z to 11480 (first estimation[36]) and 25035 (second estimation [41]) in Eqs (8)–(10). For pelagic fish and marine mammals, our estimations were based exclusively on the work of Appeltans and colleagues[36]. For fish, w was fixed to 1314 species and z to 8281 and for mammals w was fixed to 125 species and z to 8043 in Eqs (8)–(10). In all estimations, x (i.e. the species richness of a given taxonomic group) = 39 species for foraminifers, x = 86 species for euphausiids, x = 12 species for tuna/billfish, x = 27 species for oceanic sharks, x = 81 species for cetaceans and x = 36 species for pinnipeds. And y = 19609 pseudo-species richness for all estimations.

Histograms of the values assessed for each geographical square was then performed for each guild (Fig 9): (i) Protozooplankton, (ii) Metazooplankton, (iii) pelagic oceanic fish and (iv) marine mammals. The mode of each histogram, as well as the median of all values of niche saturation, were assessed to provide a global mean niche saturation value (Table 1).

## Results and discussion

Using METAL (S1 Text) we can model ecogeographic biodiversity patterns by creating a pool of theoretical pseudo-species that each has a distinct thermal optimum and breadth. Each theoretical pseudo-species can occupy a given region of the ocean so long as they can withstand local thermal fluctuations (Methods, Fig 2 and S1 Text). In each geographical cell of a gridded ocean, pseudo-species form a pseudo-community that is characterized by its richness. We found that large-scale biodiversity patterns modeled by Gaussian niches (Fig 3) were very similar to biodiversity patterns modeled by rectangular niches (r = 0.99; p<0.01; n = 9927, n* = 4)[7]; this result suggests that the shape of the niche does not alter biodiversity patterns significantly. Spatial patterns in expected biodiversity were correlated positively and strongly with mean SSTs (r = 0.89,p<0.01,n = 10578,n* = 5) and negatively and strongly with long-term (1850–2014) monthly variability in SSTs (r = -0.74,p<0.01,n = 10578,n* = 8). This joint influence of mean and variability in SST was also detected in a previous model performed at a weekly temporal resolution where both eurythermic and thermophilic species were important in generating the maximum of biodiversity that occurs in the mid-latitude regions[7, 8]. Although the Mid-Domain Effect (MDE), as defined originally in the geographical space[23], did not influence the ecogeographic biodiversity patterns in our study because the peak of biodiversity did not take place at the equator (i.e. the mid-range of the domain), we showed previously that a MDE occurring in the Euclidean space of the thermal niche affects ecogeographic biodiversity patterns [7].

We found positive correlations between expected and observed ecogeographic biodiversity patterns for foraminifers (r = 0.87,p<0.01,n = 9853,n* = 5), euphausiids (r = 0.79,p<0.01,n = 9853,n* = 7), oceanic sharks (r = 0.76,p<0.01,n = 9853,n* = 7), tuna/billfish (r = 0.82,p<0.01,n = 9853,n* = 6), and cetaceans (r = 0.68,p<0.01,n = 9853,n* = 9), while pinnipeds were negatively correlated (r = -0.82,p<0.01,n = 9853,n* = 6)[7]. The positive correlations suggest the existence of a deterministic component to how marine life is arranged[6]. As a consequence Fig 3 can be seen as a type of chessboard (Fig 4) where each geographic square has a distinct number of sub-squares, determined by the maximum number of species’ niches at saturation, and where each sub-square can hold only one species of the same trophic guild, following Gause’s principle of competitive exclusion[21]. In other words, the chessboard constrains the number of species that can co-occur in each geographical square; note that the size of a sub-square in Fig 4 is arbitrary (i.e. fixed by the number of sub-squares within a geographical square in the example) and should not be related to the size of the corresponding ecological niche. Fig 3 shows that the number of sub-squares is higher in the tropics and lower polewards. The number of actual species out of the number of sub-squares determines niche saturation in each square for a taxonomic group (Fig 4)[33].

Positive significant correlations were found between observed biodiversity patterns and mean SSTs for all species but pinnipeds (negative significant correlations): foraminifers (r = 0.92,p<0.01,n = 9853,n* = 5), euphausiids (r = 0.83,p<0.01,n = 9853,n* = 6), oceanic sharks (r = 0.83,p<0.01,n = 9853,n* = 6), tuna/billfish (r = 0.84,p<0.01,n = 9853,n* = 6), cetaceans (r = 0.56,p<0.01,n = 9853,n* = 13), pinnipeds (r = -0.87,p<0.01,n = 9853,n* = 5). The opposite significant correlations were found between observed biodiversity patterns and SST variability: foraminifers (r = -0.76,p<0.01,n = 9853,n* = 7), euphausiids (r = -0.70,p<0.01,n = 9853,n* = 8), oceanic sharks (r = -0.61,p<0.01,n = 9853,n* = 11), tuna/billfish (r = -0.71,p<0.01,n = 9853,n* = 8), cetaceans (r = -0.69,p<0.01,n = 9853,n* = 8), pinnipeds (r = 0.68,p<0.01,n = 9853,n* = 9). Similar conclusions were reached by Tittensor and colleagues[4] using SST range and slope instead of the coefficient of variation of SSTs (their Table 1). Therefore, both modelled (METAL) and observed biodiversity were significantly correlated with SST mean and variability, although the correlation was inverted for pinnipeds in comparison to other taxonomic groups.

Large-scale ecogeographic patterns in niche saturation have not been shown on a global scale before. The removal of the deterministic effect fixed by the underlying chessboard (here assessed by calculating expected biodiversity from METAL) may provide interesting clues on other mechanisms that affect the arrangement of life. We show that different ecogeographic patterns of niche saturation exist among the 6 taxonomic groups, which suggests the existence of a particular chessboard for each group that might originate from taxon-specific diversification history[42, 43](Fig 5). Although it is commonly assumed that niche saturation increases towards the equator (i) because evolutionary rates have been hypothesised to increase from cold to warm regions[29, 44] and (ii) because of the presence of strong climate-induced environmental perturbations in extra-tropical regions that limit species richness[15, 45], we found that niche saturation was highest towards the poles for all groups but fish (Fig 5). Those results show that the few sub-squares available in polar regions are frequently occupied, which also suggests that low polar biodiversity should not always be attributed to low diversification rates (origination *minus* extinction)[46, 47], but rather to smaller maximum number of species’ niches at saturation that limits local biodiversity fundamentally. When we excluded polar regions, niche saturation of all groups but mammals was higher over permanently stratified regions.

To better understand the origin of ecogeographic patterns in niche saturation, we represented niche saturation and key ecological properties as a function of mean SST above -1°C and long-term (1850–2014) monthly variability in SSTs; these last two variables are important in explaining marine biodiversity patterns (S2 Text)[4, 17]. All patterns in niche saturation and explanatory variables were significant (S1 and S2 Tables). Fig 6 shows patterns in niche saturation as a function of mean SST and long-term (1850–2014) monthly variability in SSTs. Higher niche saturation (red bullets in Fig 6) was observed towards colder regions for all taxonomic groups but oceanic sharks (Fig 6) and it coincided with (i) higher modeled pseudo-community eurythermy that provides a better resistance to climate[8], (ii) lower modeled pseudo-community thermophily, (iii) higher influence of long-term climate change on modeled biodiversity, (iv) smaller maximum expected number of species’niches and (v) low evolutionary rates as modeled from the MTE (Fig 7). For long-term (1850–2014) monthly variability in SSTs above 0.7, many taxa exhibited low niche saturation, possibly because of conditions that are too harsh [6]. A focus on saturation for SSTs ranging from 2.75°C to 30°C (excluding Polar regions) revealed a second pattern, an increase in saturation from cold-temperate to tropical waters for plankton and fish (see small panels in Fig 6). Despite having more sub-squares in the tropics, the density of occupation of the chessboard is higher in tropical than temperate zones for plankton and fish; this is probably because of greater net tropical diversification rates[46, 47] or faster species turnover in extratropical regions [48](Fig 7C, 7D and 7F; small panels).

We performed two Principal Components Analyses (PCAs) on the matrix of niche saturation [categories of mean SST and its long-term monthly variability in SSTs x 6 taxonomic groups] for mean SSTs (i) above -1°C and (ii) 2.75°C and with ecological parameters described in Fig 7 as supplementary variables (Fig 8). Plankton and marine mammals were correlated negatively with maximum species’ niches at saturation and fish were either uncorrelated (tuna/billfish) or correlated positively (Fig 8C; S3 Table). Plankton and mammals were significantly positively correlated with pseudo-community eurythermy, which suggests they may be resistant to strong thermal fluctuations (Fig 8C; S3 Table); while this result was confirmed by the positive correlation with modeled biodiversity changes between the Last Glacial Maximum (LGM) and today it contrasts with the small thermal range covered by pinnipeds (S1 Fig and S4 Text). Oceanic sharks were significantly correlated positively with pseudo-community thermophily and corresponding evolutionary rates assessed from the MTE, in contrast to tuna/billfish (Fig 8C; S3 Table). Lack of congruence among taxonomic groups may be explained by their respective diversification histories. For example, pinnipeds originate from Arctoid carnivores 25–27 Ma that were present in the cold regions of the North Pacific[49], which may also explain the contrast with other groups and the negative correlation between predicted (pseudo-species richness at saturation) and observed biodiversity (r = -0.82,p<0.01; inversed LBG). Life history strategies that are often taxon-specific may also play a role. For example, marine mammals are endotherms and well-insulated, and so they can resist thermal perturbations. Plankton are eurythermic ectotherms and large-scale dispersers. Oceanic sharks and tuna/billfish are less eurythermic and more thermophilic than plankton (S1 Fig and S4 Text) and can therefore be affected more negatively by climate change and more positively by temperature that may influence evolutionary rates[28]. All groups were monophyletic and their patterns of niche saturation were not attributable to their taxonomic level; with the exception of the Foraminifera (subphylum), most groups were orders (S4 Table).

The second PCA performed on annual SST above 2.75°C confirmed that mammals were more representative of a eurythermic community and unaffected by climate change (Fig 8D–8F and S3 Table). Plankton and fish are both more thermophilic than mammals (S1 Fig and S4 Text) and are also negatively affected by climate change and positively by evolutionary rates as assessed from the MTE. Plankton was less influenced by maximum species’ niches at saturation between 2.75°C and 30°C, but correlations remained unchanged with mammals. It therefore appears that changes in net diversification rates influence patterns in niche saturation between tropical and temperate regions where the effect of maximum species’ niche is weaker in contrast to the poles (Fig 3). Our results therefore agree with the time hypothesis[50], which stipulates that the tropics assemble more species over a longer time period because they are more stable; this is interesting because studies suggest that the tropics are both a species’ cradle (higher origination rates) and a museum (more long-term climatic stability)[46].

Finally, we used the taxonomic groups as representative of ecological guilds to assess their niche saturation in terms of absolute value rather than relative patterns; foraminifers for protozooplankton, euphausiids for metazooplankton, both tuna/billfish and oceanic sharks for pelagic fish, and both cetaceans and pinnipeds for mammals (Fig 9). Niche saturation of mammals was low (~0.5%; median = 0.67%; Table 1) in comparison to other groups (Fig 9); fish saturation exhibited a mode at ~10% (median = 7.62%), protozooplankton at ~15% (median = 14.47%) and metazooplankton at ~35–40% (median = 37.69%). A correction was applied to account for species that have not yet been discovered using two estimates: the first was mainly based upon the work of Appeltans and colleagues[36] and was applied to all guilds. The second, only used for plankton, was based upon the work of TARA[41]. While the first correction did not affect our results substantially, the second increased values of saturation for protozooplankton (Fig 9 and Table 1). After correction, our results showed an increase in saturation with diminishing organism complexity; the highest values were found for protozooplankton (or only plankton when Appeltans’ correction was applied) and the smallest was found for mammals. Our results suggest that communities can be far from saturation[15, 51] and that the total number of species on the chessboard diminishes with organismal complexity. The decrease with organism complexity can be explained by basic ecological and evolutionary processes. Endosomatic energy decreases from primary producers to higher trophic levels as a consequence of the second law of thermodynamics, diminishing the number of individuals and therefore species richness and niche saturation from producers to top predators[2, 6]; a positive relationships between number of individuals and species richness has often been proposed in theories or hypotheses developed to explain the cause of the LBG, for examples, the productivity theory [52], the Area Hypothesis [53], and the Unified Neutral Theory of Biodiversity and Biogeography [54]. In addition to diminishing the number of individuals[28], larger body size also increases generation time, which slows down evolution[28, 29]. We propose that the likelihood a taxon exhibits an ecogeographic biodiversity pattern different to the one imposed by the life chessboard is higher when its mean niche saturation is weaker; this proposition is valid in this work because correlations between expected and observed patterns are smaller (cetaceans) or even opposite (pinnipeds) for marine mammals.

Hutchinson[20] recognized that it is impossible to use all niche dimensions and it is therefore important to select those that control a large part of the spatial distribution of species. Temperature is a parameter frequently identified as being instrumental in explaining species, community, ecosystem and biome distribution in global-scale studies, influencing the biology and ecology of all species[4, 55–57]. We therefore used a one-dimensional (thermal) niche to reconstruct biodiversity[7, 8] at a macro-scale. While we consider our approach to be valid at a macro-scale, we also anticipate that future versions of our framework will incorporate more ecological dimensions to provide more precise estimations of biodiversity and niche saturation at regional or local scales. At smaller scales, environmental parameters such as bathymetry, nutrients (e.g. nitrate, phosphate, silicate, iron), and oxygen concentration are likely to play an important role in shaping the arrangement of some ecological guilds or taxonomic groups[57–59]. Resource availability is also expected to be important as it controls the number of individuals, which in turn should promote more species[54, 60, 61]. However at a large scale, primary production (the unit of endosomatic energy produced per unit of time and space), is not correlated positively with marine biodiversity[6, 18], suggesting that resource may not be the primary cause of the LBG.

## Conclusions

Many hypotheses have been proposed to explain the primary cause of the latitudinal patterns in biodiversity[2, 3, 62]. While some authors have proposed that the LBG is due to the larger area of the tropical belts[61], the neutral model of biodiversity and biogeography[54], the mid-domain effect[63], or evolutionary explanations[64] are particularly popular. Perhaps the most compelling hypotheses have been those that invoke an environmental control of biodiversity such as environmental stability or energy availability[4, 65, 66]. As we have indicated however, temperature has been often suggested to explain large-scale patterns in the distribution of marine organisms[4, 17], although the mechanisms by which this parameter acts to create latitudinal gradients in marine biodiversity has remained elusive[4]. The joint examination of biodiversity and niche saturation on a global scale in this study has provided interesting clues to the mechanisms underlying this pattern. Our results show that oceanic biodiversity is spatially constrained by an underlying chessboard-like structure that exerts a strong deterministic geographic effect on the macro-scale organization of marine eukaryotes by fixing the maximum number of species that can establish regionally; we exclude for the moment prokaryotes for which our framework has not been tested. We use the analogy of a chessboard to characterize this mathematical effect (Fig 4). Each square on the chessboard represents a geographical cell and the potential species that may occupy a square at saturation determine the sub-squares, which may be filled by speciation and immigration or purged by extinction (Fig 4). The deterministic effect imposed by the chessboard is strongest currently between the poles and other oceanic regions and weakest between temperate and permanently stratified biomes because the latter have smaller differences in biodiversity (Fig 3).

We propose that the presence of an underlying chessboard creates the establishment of the LBG for two reasons. First, it constraints large-scale oceanic patterns of biodiversity by determining a maximum number of species that can establish by speciation or immigration in a given region. The constraints the chessboard imposes on biodiversity may be rapidly perceptible because clade diversification takes place relatively quickly initially on a geological time scale[67]. Plankton and oceanic fish, having virtually unlimited dispersal, may rapidly conform to the mathematical structure imposed by the chessboard. Second, many clades should exhibit a LBG because their probability of emergence should be higher in the tropics where there are more available niches and palaeontological data have provided compelling evidence of greater rates of origination for tropical clades[68]. The higher degree of saturation found in the tropics for plankton and fish suggest that higher diversification rates or slower species turnover may originate from higher speciation rates, lower extinction rates or both [46, 48, 64].

Our study also suggests that the diversification processes (e.g. place of origination and time of emergence) may significantly blur the patterns of biodiversity imposed by the chessboard of life. Pinnipeds for example, that originated in extratropical regions of the North Pacific 25–27 Ma ago[49], do not exhibit an expected LGB. Nevertheless, pinniped biodiversity remains probably constrained in term of species number. Indeed, cetaceans, which are older but also originated in warmer regions[69] where the number of niches is higher, have a greater biodiversity than pinnipeds. Providing that pinnipeds have not evolved life history traits or anatomical structures that prevent them to be eurygraph, the diversification of this clade may continue and eventually lead to a more global-scale distribution that could be constrained by the chessboard of life. Because saturation is more important when biocomplexity diminishes, it follows necessarily, that the deterministic effect induced by the chessboard of life is likely to be more prominent for simpler (e.g. plankton) than for more complex taxonomic groups (e.g. mammals) of eukaryotes.

The rate of net diversification is a fundamental parameter because it determines the degree of niche occupancy in a square and the emerging life history traits and strategies make the chessboard specific to each clade; this explains the lack of universality in ecogeographic patterns (i.e. lack of congruence of the biodiversity among taxonomic groups). In a previous study, we have shown that climate-induced biogeographical shifts may alter chessboard’s sub-squares and therefore the species associated with them[9, 66]. When a sub-square is occupied by a species they are both expected to move equatorwards in the case of global cooling, reinforcing the LBG slope, and to move polewards in the case of global warming, diminishing the LBG slope (Fig 4). This movement makes the chessboard highly dynamic on time scales from decades to millions of years. By merging evolutionary, ecological and mathematical mechanisms, our theory lies at the intersection of ecological and historical biogeography. Our theory, which we consider should also apply to the benthic domain and the terrestrial realm, explains why many taxonomic groups in the oceans exhibit a LBG, and it provides a comprehensive explanation why some clades may have different ecogeographic patterns.

## Supporting information

S1 Fig Average observed mean species richness as a function of annual SST for six taxonomic groups. The observed mean thermal optimum (T_o_) and mean thermal range (E_o_) of the taxonomic group is indicated. **(A)** foraminifers, **(B)** euphausiids, **(C)** oceanic sharks, **(D)** tuna/billfish, **(E)** cetaceans and **(F)** pinnipeds.
(TIF)

S1 Table Summary of the results of the 2-order polynomial regression between the degree of niche saturation of each taxonomic group and both mean annual SST (between -1°C and 30°C) and annual SST variability. See Fig 6. All probabilities were significant, i.e. lower than 0.01. For all regressions, degrees of freedom were 179. Adjusted R^2^ consider the reduction of the degree of freedom when a variable is added to the regression. RMSE: Root Mean Squared Error; the smaller the value, the better the fit of the regression.
(DOCX)

S2 Table Summary of the results of the 2-order polynomial regression between the value of each explanative variable and both mean annual SST (between -1°C and 30°C) and annual SST variability. See Fig 7. All probabilities were significant, i.e. lower than 0.01. RMSE: Root Mean Squared Error. Higher RMSE are observed for LGM/today and mid-Pliocene/today changes because RMSE has the same unit as the response variable, which has an interval of variation higher than other variables.
(DOCX)

S3 Table Table showing the correlations between changes in niche saturation of the six taxonomic groups and the explanatory variables according to mean SST and SST variability. Correlations in the lower triangle are based on mean SST ranging from -1°C to 30°C (185 degrees of freedom for all variables but LGM (157)) and those in the upper triangle are from 2.75°C to 30°C (149 degrees of freedom for all variables). Bold values are significant at p<0.01. Foram: foraminifers, Eupha: euphausiids, Shark: oceanic sharks, Tuna: tuna/billfish, Ceta: Cetaceans, Pinni:pinnipeds. Supplementary variables, indicated by a black bullet, are degree of eurythermy of the pseudo-community for each unit of mean SST and coefficient of variation of SST (Eury; see Fig 3) and similarly for the degree of pseudo-community thermophily (Thermo), LGM/today quantitative biodiversity changes (LGM), mid-Pliocene/today quantitative biodiversity changes (Plio),theoretical number of niche/species at saturation (theoS) and mass-corrected rate of evolution (Evol).
(DOCX)

S4 Table Classification of each taxonomic group considered in this study and its phylogeny. (DOCX)

S1 Text The MacroEcological theory on the arrangement of life. (DOCX)

S2 Text Strength and limitation of the use of a single parameter (temperature). (DOCX)

S3 Text Correction of the estimations of species richness for all taxonomic groups. (DOCX)

S4 Text Assessment of the mean thermal optimum and range of all taxonomic groups. (DOCX)

S1 Data Modelled species richness from METAL and estimated niche saturation of foraminifers, euphausiids, oceanic sharks, tuna and billfish, cetaceans and pinnipeds. (XLSX)

## References

[1] Gaston KJ. Global patterns in biodiversity. Nature. 2000;405:220–7. doi: 10.1038/35012228 10821282 10.1038/35012228

[2] Lomolino MV, Riddle BR, Brown JH. Biogeography. 3 ed. Sunderland: Sinauer Associates, Inc; 2006 845 p.

[3] Rohde K. Latitudinal gradients in species diversity: the search for the primary cause. Oikos. 1992;65:514–27.

[4] Tittensor DT, Mora C, Jetz W, Lotze HK, Ricard D, Berghe EV, et al Global patterns and predictors of marine biodiversity across taxa. Nature. 2010;466:1098–101. doi: 10.1038/nature09329 20668450 10.1038/nature09329

[5] Hutchinson GE. Concluding remarks. Cold Spring Harbor Symposium Quantitative Biology. 1957;22:415–27.

[6] Beaugrand G. Marine biodiversity, climatic variability and global change. Oceans E, editor. London: Routledge; 2015 474 p.

[7] Beaugrand G, Rombouts I, Kirby RR. Towards an understanding of the pattern of biodiversity in the oceans. Global Ecology and Biogeography. 2013;22:440–9.

[8] Beaugrand G, Edwards M, Raybaud V, Goberville E, Kirby RR. Future vulnerability of marine biodiversity compared with contemporary and past changes. Nature Climate Change. 2015;5:695–701. doi: 10.1038/NCLIMATE2650

[9] Beaugrand G, Goberville E, Luczak C, Kirby RR. Marine biological shifts and climate. Proceedings of the Royal Society B: Biological Sciences. 2014;281:20133350 doi: 10.1098/rspb.2013.3350 24718760 10.1098/rspb.2013.3350 PMC3996605

[10] Beaugrand G, Kirby RR. How do marine species respond to climate change? Theories and observations. Annual Review of Marine Sciences. 2018;10:169–97. 10.1146/annurev-marine-121916-063304 29298137

[11] Beaugrand G, Mackas D, Goberville E. Applying the concept of the ecological niche and a macroecological approach to understand how climate influences zooplankton: advantages, assumptions, limitations and requirements. Progress in Oceanography. 2013;111:75–90. doi: 10.1016/j.pocean.2012.11.002

[12] Beaugrand G, Kirby RR. Quasi-deterministic responses of marine species to climate change. Climate Research. 2016;69(2):117–28. doi: 10.3354/cr01398

[13] Beaugrand G, Kirby RR. Spatial changes in the sensitivity of Atlantic cod to climate-driven effects in the plankton. Climate research. 2010;41:15–9.

[14] Beaugrand G. Theoretical basis for predicting climate-induced abrupt shifts in the oceans. Philosophical Tansactions of the Royal Society B: Biological Sciences. 2014;370 20130264. doi: 10.1098/rstb.2013.0264

[15] Rohde K. Nonequilibrium ecology. Cambridge: Cambridge University Press; 2005.

[16] Smith TM, Reynolds RW, Peterson TC, Lawrimore J. Improvements to NOAA's Historical Merged Land-Ocean Surface Temperature Analysis (1880–2006). Journal of Climate. 2008;21:2283–96.

[17] Beaugrand G, Edwards M, Legendre L. Marine biodiversity, ecosystem functioning and the carbon cycles. Proceedings of the National Academy of Sciences of the USA. 2010;107:10120–4. doi: 10.1073/pnas.0913855107 20479247 10.1073/pnas.0913855107 PMC2890445

[18] Rombouts I, Beaugrand G, Ibañez F, Gasparini S, Chiba S, Legendre L. A multivariate approach to large-scale variation in marine planktonic copepod diversity and its environmental correlates. Limnology and Oceanography. 2010;55:2219–29.

[19] Rombouts I, Beaugrand G, Ibañez F, Gasparini S, Chiba S, Legendre L. Global latitudinal variations in marine copepod diversity and environmental factors. Proceedings of the Royal Society B. 2009;276:3053–62. doi: 10.1098/rspb.2009.0742 19515670 10.1098/rspb.2009.0742 PMC2817135

[20] Hutchinson GE. An introduction to population ecology. New Haven: Yale University Press; 1978 260 p.

[21] Gause GF. The struggle for coexistence. Baltimore: MD: Williams and Wilkins; 1934.

[22] Ter Braak CJF. Unimodal models to relate species to environment. Wageningen: DLO-Agricultural Mathematics Group; 1996 266 p.

[23] Colwell RK, Lees DC. The mid-domain effect: geometric constraints on the geography of species richness. Trends in Ecology and Evolution. 2000;15:70–6. 10652559 10.1016/s0169-5347(99)01767-x

[24] Gross K, Snyder-Beattie A. A General, Synthetic Model for Predicting Biodiversity Gradients from Environmental Geometry. The American Naturalist. 2016;188:E85–E97. doi: 10.1086/688171 27622881 10.1086/688171

[25] Tomasovych A, Jablonski D. Decoupling of latitudinal gradients in species and genus geographic range size: a signature of clade range expansion. Global Ecology and Biogeography. 2016;26:288–303. doi: 10.1111/geb.12533

[26] Lam NSN. Spatial interpolation methods: a review. American cartography. 1983;10:129–49.

[27] Hawkins BA, Diniz-Filho JAF, Bini LM, Araujo MB, Field R, Hortal J, et al Metabolic theory and diversity gradients: where do we go from here? Ecology. 2007;88:1898–902. 17824418 10.1890/06-2141.1

[28] Brown JH, Gillooly JF, Allen AP, Savage VM, West GB. Toward a metabolic theory of ecology. Ecology. 2004;85:1771–89.

[29] Gillooly J, Allen AP, West GB, Brown JH. The rate of DNA evolution: effects of body size and temperature on the molecular clock. Proceedings of the National Academy of Sciences of the United States of America. 2005;102:140–5. doi: 10.1073/pnas.0407735101 15618408 10.1073/pnas.0407735101 PMC544068

[30] Beaugrand G, Edwards M, Brander K, Luczak C, Ibañez F. Causes and projections of abrupt climate-driven ecosystem shifts in the North Atlantic. Ecology Letters. 2008;11:1157–68. doi: 10.1111/j.1461-0248.2008.01218.x 18647332 10.1111/j.1461-0248.2008.01218.x

[31] Helaouët P, Beaugrand G, Reid PC. Macrophysiology of *Calanus finmarchicus* in the North Atlantic Ocean. Progress in Oceanography. 2011;91:217–28. doi: 10.1016/j.pocean.2010.11.003

[32] Beaugrand G, Ibañez F. Spatial dependence of pelagic diversity in the North Atlantic Ocean. Marine Ecology Progress Series. 2002;232:197–211.

[33] R.H. M, E.O. W. An equilibrium theory of insular zoogeography. Evolution 1963 17:373–86.

[34] Boeuf G. Marine biodiversity characteristics. Comptes Rendus Biologies. 2014;334:435–40. 10.1016/j.crvi.2011.02.009 21640952

[35] Mora C, Tittensor DP, Adl S, Simpson AGB, Worm B. How Many Species Are There on Earth and in the Ocean? PLoS Biology. 2011;9:e1001127 doi: 10.1371/journal.pbio.1001127 21886479 10.1371/journal.pbio.1001127 PMC3160336

[36] Appeltans W, Ahyong Shane T, Anderson G, Angel Martin V, Artois T, Bailly N, et al The magnitude of global marine species biodiversity. Current Biology. 2012;22:2189–202. doi: 10.1016/j.cub.2012.09.036 23159596 10.1016/j.cub.2012.09.036

[37] Beaugrand G, Ibanez F. Monitoring marine plankton ecosystems (2): long-term changes in North Sea calanoid copepods in relation to hydro-meteorological variability. Marine Ecology Progress Series. 2004;284:35–47.

[38] Legendre P, Legendre L. Numerical Ecology. 2 ed. Amsterdam: Elsevier Science B.V; 1998 853 p.

[39] Beaugrand G. Plankton biodiversity and biogeography In: Castellani C, Edwards M, editors. Marine Plankton. Oxford: Oxford University Press; 2017 p. 12–23.

[40] Froese R, Pauly D. FishBase. World Wide Web electronic publication 2017;www.fishbase.org.

[41] de Vargas C, Audic S, Henry N, Decelle J, Mahé F, Logares R, et al Eukaryotic plankton diversity in the sunlit ocean. Science. 2015;348(6237). doi: 10.1126/science.1261605 25999516 10.1126/science.1261605

[42] Benton MJ. Diversification and extinction in the history of life. Science. 1995;268:52–8. 7701342 10.1126/science.7701342

[43] Morlon H. Phylogenetic approaches for studying diversification. Ecology Letters. 2014;17:508–25. doi: 10.1111/ele.12251 24533923 10.1111/ele.12251

[44] Machac A, Zrzavy J, Smrckova J, Storch D. Temperature dependence of evolutionary diversification: differences between two contrasting model taxa support the metabolic theory of ecology. Journal of Evolutionary Biology. 2012;25:2449–56. doi: 10.1111/jeb.12019 23116407 10.1111/jeb.12019

[45] Crame JA. Pattern and proceses in marine biogeography: a view from the poles In: Lomolino MV, Heaney LR, editors. Fontiers of biogeography I: new directions in the geography of nature. Sunderland: MA: Sinauer Associates; 2004 p. 272–92.

[46] Jablonski D, Roy K, Valentine JW. Out of the Tropics: evolutionary dynamics of the latitudinal diversity gradient. Science. 2006;314:102–6. doi: 10.1126/science.1130880 17023653 10.1126/science.1130880

[47] Dowle EJ, Morgan-Richards M, Trewick SA. Molecular evolution and the latitudinal biodiversity gradient. Heredity. 2013;110:501–10. doi: 10.1038/hdy.2013.4 23486082 10.1038/hdy.2013.4 PMC3656639

[48] Weir JT, Schluter D. The latitudinal gradient in recent speciation and extinction rates of birds and mammals. Science. 2007;315:1574–6. doi: 10.1126/science.1135590 17363673 10.1126/science.1135590

[49] Berta A, Adam P. The evolutionary biology of pinnipeds In: de Buffrenil V, Mazin J-M, editors. Secondary adaptation of tetrapods to life in the water Munchen Germany: Verlag Dr Frederich Pfeil; 2001 p. 235–60.

[50] Pianka ER. Latitudinal gradients in species diversity: a review of concepts. The American Naturalist. 1966;100:33–46.

[51] Walker TD, Valentine JW. Equilibrium models of evolutionary diversity and the number of empty niches. The American Naturalist. 1984;124:887–99.

[52] Hawkins BA, Porter EE. Does herbivore diversity depend on plant diversity? The case of California butterflies. The American Naturalist. 2003;161:40–9. doi: 10.1086/345479 12650461 10.1086/345479

[53] MacArthur RH, Wilson EO. The theory of island biogeography. Princeton Princeton University Press; 1967.

[54] Hubbell SP. The unified neutral theoy of biodiversity and biogeography. Princeton: Princeton University Press; 2001.

[55] Burrows MT, Schoeman DS, Buckley LB, Moore P, Poloczanska ES, Brander KM, et al The pace of shifting climate in marine and terrestrial ecosystems. Science. 2011;334:652–5. doi: 10.1126/science.1210288 22053045 10.1126/science.1210288

[56] Sunday JM, Bates AE, Dulvy NK. Thermal tolerance and the global redistribution of animals. Nature Climate Change. 2012:1–5. doi: 10.1038/NCLIMATE1539

[57] Reygondeau G, Longhurst A, Beaugrand G, Martinez E, Antoine D, Maury O. Toward Dynamic Biogeochemical Provinces. Global Biogeochemical cycles. 2013;27:1046–58.

[58] Sarmiento JL, Gruber N. Ocean biogeochemical dynamics. Princeton and Oxford: Princeton University Press; 2006 503 p.

[59] Longhurst A. Ecological geography of the Sea. London: Academic Press; 1998 390 p.

[60] Wright DH. Species-energy theory: an extension of species-area theory. Oikos. 1983;41:496–506.

[61] Rosenzweig ML. Species diversity in space and time. Cambridge: Cambridge University Press; 1995 436 p.

[62] Rosenzweig ML, Sandlin EA. Species diversity and latitudes: listening to area's signal. Oikos. 1997;80:172–6.

[63] Colwell RK, Hurtt GC. Nonbiological gradients in species richness and a spurious rapoport effect. The American Naturalist. 1994;144:570–95.

[64] Mittelbach GG, Schemske DW, Cornell HV, Allen AP, Brown JM, Bush MB, et al Evolution and the latitudinal diversity gradient: speciation, extinction and biogeography. Ecology Letters. 2007;10:315–31. doi: 10.1111/j.1461-0248.2007.01020.x 17355570 10.1111/j.1461-0248.2007.01020.x

[65] Rutherford S, D'Hondt S, Prell W. Environmental controls on the geographic distribution of zooplankton diversity. Nature. 1999;400:749–53.

[66] Beaugrand G, Reid PC, Ibañez F, Lindley JA, Edwards M. Reorganisation of North Atlantic marine copepod biodiversity and climate. Science. 2002;296:1692–4. doi: 10.1126/science.1071329 12040196 10.1126/science.1071329

[67] Rabosky DL, Hurlbert AH. Species richness at continental sclaes is dominated by ecological limits. The American Naturalist 2015 185:572–83. doi: 10.1086/680850 25905501 10.1086/680850

[68] Wiens JJ, Donoghue MJ. Historical biogeography, ecology and species richness. Trends in Ecology and Evolution. 2004;19:639–44. doi: 10.1016/j.tree.2004.09.011 16701326 10.1016/j.tree.2004.09.011

[69] Milinkovitch MC, Meyer A, Powell JR. Phylogeny of all major groups of Cetaceans based on DNA sequences from three mitochondrial genes. Molecular Biology and Evolution. 1994;11:939–48. doi: 10.1093/oxfordjournals.molbev.a040164 7755710 10.1093/oxfordjournals.molbev.a040164

