# Supplementary figures and images for: Marine biodiversity and the chessboard of life
Source: PLoS One. 2018 Mar 22;13(3):e0194006. doi: 10.1371/journal.pone.0194006 (PMC5864006; doi:10.1371/journal.pone.0194006)

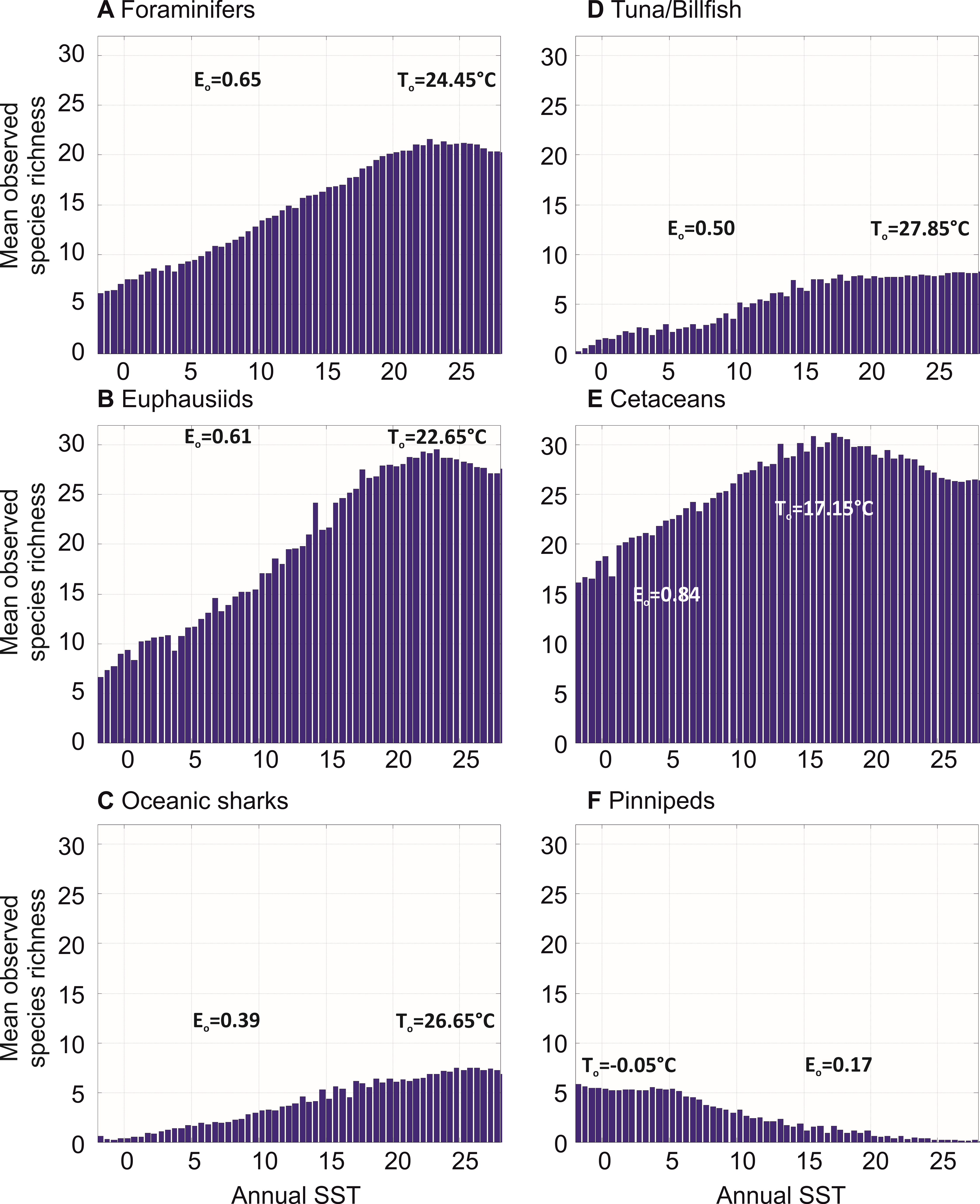

Supplement: S1 Fig — The observed mean thermal optimum (To) and mean thermal range (Eo) of the taxonomic group is indicated. (A) foraminifers, (B) euphausiids, (C) oceanic sharks, (D) tuna/billfish, (E) cetaceans and (F) pinnipeds. (TIF) [file pone.0194006.s001.tif]
