# Supplementary material for: Marine biodiversity and the chessboard of life
Source: PLoS One. 2018 Mar 22;13(3):e0194006. doi: 10.1371/journal.pone.0194006 (PMC5864006; doi:10.1371/journal.pone.0194006)
Supplement: S1 Table — See Fig 6. All probabilities were significant, i.e. lower than 0.01. For all regressions, degrees of freedom were 179. Adjusted R2 consider the reduction of the degree of freedom when a variable is added to the regression. RMSE: Root Mean Squared Error; the smaller the value, the better the fit of the regression. (DOCX) [file pone.0194006.s002.docx]

**S1 Table | Summary of the results of the 2-order polynomial regression between the degree of niche saturation of each taxonomic group and both mean annual SST (between -1°C and 30°C) and annual SST variability**. See Fig. 6. All probabilities were significant, i.e. lower than 0.01. For all regressions, degrees of freedom were 179. Adjusted R² consider the reduction of the degree of freedom when a variable is added to the regression. RMSE: Root Mean Squared Error; the smaller the value, the better the fit of the regression.

| Taxonomic group | R | Adjusted R² | RMSE |
| --- | --- | --- | --- |
| Foraminifers | 0.99 | 0.97 | 0.0002 |
| Euphausiids | 0.96 | 0.93 | 0.0003 |
| Oceanic sharks | 0.94 | 0.88 | 0.0001 |
| Tuna/billfish | 0.67 | 0.44 | 0.0002 |
| Cetaceans | 0.98 | 0.96 | 0.0006 |
| Pinnipeds | 0.99 | 0.98 | 0.0001 |
