# Supplementary material for: Marine biodiversity and the chessboard of life
Source: PLoS One. 2018 Mar 22;13(3):e0194006. doi: 10.1371/journal.pone.0194006 (PMC5864006; doi:10.1371/journal.pone.0194006)
Supplement: S2 Table — See Fig 7. All probabilities were significant, i.e. lower than 0.01. RMSE: Root Mean Squared Error. Higher RMSE are observed for LGM/today and mid-Pliocene/today changes because RMSE has the same unit as the response variable, which has an interval of variation higher than other variables. (DOCX) [file pone.0194006.s003.docx]

**S2 Table | Summary of the results of the 2-order polynomial regression between the value of each explanative variable and both mean annual SST (between -1°C and 30°C) and annual SST variability.** See Fig. 7. All probabilities were significant, i.e. lower than 0.01. RMSE: Root Mean Squared Error. Higher RMSE are observed for LGM/today and mid-Pliocene/today changes because RMSE has the same unit as the response variable, which has an interval of variation higher than other variables.

| Explanatory variables | Degree of freedom | R | Adjusted R² | RMSE |
| --- | --- | --- | --- | --- |
| Eurythermy | 179 | 0.96 | 0.91 | 0.0179 |
| Thermophily | 179 | 0.99 | 0.99 | 0.1397 |
| LGM/today changes | 151 | 0.88 | 0.76 | 4.2151 |
| Mid-Pliocene/today changes | 179 | 0.93 | 0.86 | 2.4632 |
| Niche/species at saturation | 179 | 0.99 | 0.98 | 0.0379 |
| Mass-corrected rates of evolution | 179 | 0.99 | 0.99 | 0.0310 |
