# Supplementary material for: Marine biodiversity and the chessboard of life
Source: PLoS One. 2018 Mar 22;13(3):e0194006. doi: 10.1371/journal.pone.0194006 (PMC5864006; doi:10.1371/journal.pone.0194006)
Supplement: S3 Table — Correlations in the lower triangle are based on mean SST ranging from -1°C to 30°C (185 degrees of freedom for all variables but LGM (157)) and those in the upper triangle are from 2.75°C to 30°C (149 degrees of freedom for all variables). Bold values are significant at p<0.01. Foram: foraminifers, Eupha: euphausiids, Shark: oceanic sharks, Tuna: tuna/billfish, Ceta: Cetaceans, Pinni:pinnipeds. Supplementary variables, indicated by a black bullet, are degree of eurythermy of the pseudo-community for each unit of mean SST and coefficient of variation of SST (Eury; see Fig 3) and similarly for the degree of pseudo-community thermophily (Thermo), LGM/today quantitative biodiversity changes (LGM), mid-Pliocene/today quantitative biodiversity changes (Plio),theoretical number of niche/species at saturation (theoS) and mass-corrected rate of evolution (Evol). (DOCX) [file pone.0194006.s004.docx]

**S3 Table | Table showing the correlations between changes in niche saturation of the six taxonomic groups and the explanatory variables according to mean SST and SST variability.** Correlations in the lower triangle are based on mean SST ranging from -1°C to 30°C (185 degrees of freedom for all variables but LGM (157)) and those in the upper triangle are from 2.75°C to 30°C (149 degrees of freedom for all variables). Bold values are significant at p<0.01. Foram: foraminifers, Eupha: euphausiids, Shark: oceanic sharks, Tuna: tuna/billfish, Ceta: Cetaceans, Pinni:pinnipeds. Supplementary variables, indicated by a black bullet, are degree of eurythermy of the pseudo-community for each unit of mean SST and coefficient of variation of SST (Eury; see Fig. 3) and similarly for the degree of pseudo-community thermophily (Thermo), LGM/today quantitative biodiversity changes (LGM), mid-Pliocene/today quantitative biodiversity changes (Plio),theoretical number of niche/species at saturation (theoS) and mass-corrected rate of evolution (Evol).

|  | **Foram** | **Euph** | **Shark** | **Tuna** | **Ceta** | **Pinni** | **Eury** | **Thermo** | **LGM** | **Plio** | **TheoS** | **Evol** |
| --- | --- | --- | --- | --- | --- | --- | --- | --- | --- | --- | --- | --- |
| **Foram** |  | **0.79** | **0.34** | **0.50** | 0.12 | -0.06 | 0.10 | **0.44** | **-0.27** | **-0.61** | -0.07 | **0.43** |
| **Euph** | **0.93** |  | **0.60** | **0.62** | -0.21 | **-0.31** | -0.21 | **0.65** | **-0.49** | **-0.59** | **0.24** | **0.64** |
| **Shark** | **-0.39** | **-0.27** |  | **0.68** | **-0.75** | **-0.75** | **-0.77** | **0.92** | **-0.73** | **-0.75** | **0.77** | **0.91** |
| **Tuna** | **0.25** | **0.39** | **0.33** |  | **-0.35** | **-0.46** | **-0.43** | **0.64** | **-0.48** | **-0.59** | **0.47** | **0.63** |
| **Ceta** | **0.96** | **0.95** | **-0.49** | **0.25** |  | **0.92** | **0.94** | **-0.81** | **0.79** | **0.57** | **-0.94** | **-0.82** |
| **Pinni** | **0.95** | **0.85** | **-0.56** | **0.16** | **0.95** |  | **0.96** | **-0.87** | **0.82** | **0.69** | **-0.97** | **-0.88** |
| **Eury** | **0.75** | **0.62** | **-0.74** | 0.03 | **0.76** | **0.84** |  | **-0.82** | **0.77** | **0.62** | **-0.97** | **-0.83** |
| **Thermo** | **-0.47** | **-0.35** | **0.93** | **0.24** | **-0.58** | **-0.67** | **-0.83** |  | **-0.83** | **-0.87** | **0.82** | **0.99** |
| **LGM** | -0.01 | **-0.26** | **-0.75** | **-0.44** | **0.72** | **0.78** | **0.69** | **-0.85** |  | **0.66** | **-0.80** | **-0.84** |
| **Plio** | -0.00 | 0.04 | **-0.65** | **-0.22** | **0.19** | **0.19** | **0.34** | **-0.70** | **0.67** |  | **-0.59** | **-0.87** |
| **TheoS** | **-0.86** | **-0.71** | **0.71** | -0.04 | **-0.85** | **-0.94** | **-0.96** | **0.80** | **-0.76** | **-0.27** |  | **0.83** |
| **Evol** | **-0.49** | **-0.36** | **0.92** | **0.22** | **-0.59** | **-0.69** | **-0.85** | **0.99** | **-0.85** | **-0.68** | **0.82** |  |
