# Supplementary material for: Marine biodiversity and the chessboard of life
Source: PLoS One. 2018 Mar 22;13(3):e0194006. doi: 10.1371/journal.pone.0194006 (PMC5864006; doi:10.1371/journal.pone.0194006)
Supplement: S4 Table — (DOCX) [file pone.0194006.s005.docx]

**S4** **Table** | **Classification of each taxonomic group considered in this study and its phylogeny.**

| **Taxonomic groups** | **Domain/Phylum** | **Level of classification** | **Name of classification** | **Monophyly** | **Reference** |
| --- | --- | --- | --- | --- | --- |
| Foraminifers | Eukaryota/Retaria | Subphylum | Foraminifera | Yes | [[1](#_ENREF_1)] |
| Euphausiids | Animalia/Arthropoda | Order | Euphausiacea | Yes | [[2](#_ENREF_2)] |
| Billfish/tuna | Animalia/Chordata | Suborder | Scombroidei | Yes | [[3](#_ENREF_3)] |
| Oceanic sharks | Animalia/Chordata | Superorder | Selachimorpha | Yes | [[4](#_ENREF_4)] |
| Cetaceans | Animalia/Chordata | Infraorder | Cetacea | Yes | [[5](#_ENREF_5)] |
| Pinnipeds | Animalia/Chordata | Suborder | Caniformia | Yes | [[6](#_ENREF_6)] |

**References**

1. Burki F, Pawlowski J. Monophyly of Rhizaria and Multigene Phylogeny of Unicellular Bikonts. Molecular Biology and Evolution. 2006;23:1922-30.

2. Jarman SN. The evolutionary history of krill inferred from nuclear large subunit rDNA sequence analysis. Biological Journal of the Linnean Society. 2001;73:199-212.

3. Dickson KA, Graham JB. Evolution and Consequences of Endothermy in Fishes. Physiological and Biochemical Zoology. 2004;77:998-1018.

4. Vélez-Zuazo X, Agnarsson I. Shark tales: A molecular species-level phylogeny of sharks (Selachimorpha, Chondrichthyes). Molecular Phylogenetics and Evolution. 2011;58:207-17.

5. Milinkovitch MC, Meyer A, Powell JR. Phylogeny of all major groups of Cetaceans based on DNA sequences from three mitochondrial genes. Molecular Biology and Evolution. 1994;11:939-48.

6. Arnason U, Gullberg A, Janke A, Kullberg M, Lehman N, Petrov EA, et al. Pinniped phylogeny and a new hypothesis for their origin and dispersal. Molecular Phylogenetics and Evolution. 2006;41:345-54.
