# Supplementary material for: Marine biodiversity and the chessboard of life
Source: PLoS One. 2018 Mar 22;13(3):e0194006. doi: 10.1371/journal.pone.0194006 (PMC5864006; doi:10.1371/journal.pone.0194006)
Supplement: S1 Text — (DOCX) [file pone.0194006.s006.docx]

**S1 Text | The MacroEcological Theory on the Arrangement of Life**

The MacroEcological Theory on the Arrangement of Life (METAL)[[1-4](#_ENREF_1)] is a theory that explains how life is arranged in the sea and how changing environmental conditions alter biological arrangements in space and time at different organisational levels (e.g. species, community, ecosystem), allowing precise predictions to be tested. Recently, we have theoretically investigated palaeo (mid-Pliocene and Last Glacial Maximum or LGM), contemporaneous (1960-2013) and future (2080-2100) changes in biodiversity in the context of global climate change to evaluate the sensitivity and vulnerability of biodiversity to climate change[[3](#_ENREF_3)]. This theoretical work has revealed that climate change may rapidly alter marine biodiversity over large oceanic regions and that the intensity of this reorganisation will depend on the magnitude of warming. If global warming is small (RCP2.6), the study has shown that biological changes would reflect 15.5% of the amount of change seen between the LGM and the present day, or 25.3% of the amount of change observed between the mid-Pliocene and today; neither are that different to annual variability (1960-2013) and so it may be benign overall. If warming is moderate (RCP4.5), changes in marine biodiversity will be three-times more extensive and at least twice as strong in magnitude than changes observed over the last 50 years. If global warming is severe (RCP6.0 and 8.5), between 50 and 70% of the global ocean will experience a change in marine biodiversity equivalent to, or higher than, that experienced between the LGM/mid-Pliocene and today emphasizing that climate warming will have a major effect on marine biodiversity. This METAL model has been used here (i) to reconstruct ecogeographical biodiversity patterns (therefore pseudo-species richness at saturation), (ii) to calculate the degree of thermophily and eurythermy of pseudo-species and (iii) to estimate the implications of long-term climate change on biodiversity.

More information on the METAL theory and its applications can be found in <http://metaltheory.weebly.com/>.

**References**

1. Beaugrand G, Goberville E, Luczak C, Kirby RR. Marine biological shifts and climate. Proceedings of the Royal Society B: Biological Sciences. 2014;281:20133350. doi: 10.1098/rspb.2013.3350.

2. Beaugrand G. Theoretical basis for predicting climate-induced abrupt shifts in the oceans. Philosophical Tansactions of the Royal Society B: Biological Sciences. 2014;370 20130264. doi: 10.1098/rstb.2013.0264.

3. Beaugrand G, Edwards M, Raybaud V, Goberville E, Kirby RR. Future vulnerability of marine biodiversity compared with contemporary and past changes. Nature Climate Change. 2015;5:695-701. doi: 10.1038/NCLIMATE2650.

4. Beaugrand G. Marine biodiversity, climatic variability and global change. Oceans E, editor. London: Routledge; 2015. 474 p.
