# Supplementary material for: Marine biodiversity and the chessboard of life
Source: PLoS One. 2018 Mar 22;13(3):e0194006. doi: 10.1371/journal.pone.0194006 (PMC5864006; doi:10.1371/journal.pone.0194006)
Supplement: S2 Text — (DOCX) [file pone.0194006.s007.docx]

**S2 Text | Strength and limitation of the use of a single parameter (temperature)**

The ecological niche is multidimensional. Unfortunately, as recognized early by Hutchinson[[1](#_ENREF_1)], it is impossible to use all niche dimensions and so it is important to select a few that control a large part of the spatial distribution of species. The climate variability hypothesis states that the latitudinal range of species is primarily determined by their thermal tolerance[[2](#_ENREF_2)]. In the marine realm, temperature is a key variable because i) it is the result of many hydro-climatic processes[[3](#_ENREF_3)] and ii) it exerts an effect on many fundamental biological and ecological processes[[4](#_ENREF_4)]. We therefore assume here that the main driver by which atmospheric forcing may affect biological communities is sea temperature because many studies showed that this parameter has a cardinal influence on species physiology, biology and ecology[[5-7](#_ENREF_5)]. Temperature controls all biological processes from the molecular to the cell and the organism levels[[6](#_ENREF_6)]. Temperature alters growth, reproduction, mortality, the behaviour of organisms at the species level and biotic interactions (positive or negative) at the community level[[8](#_ENREF_8), [9](#_ENREF_9)]. At a global scale, temperature patterns largely determine the location of biogeographic provinces and biomes and modulate the ecological services such as food production and carbon sequestration that marine ecosystems provide to humanity[[10](#_ENREF_10), [11](#_ENREF_11)]. It is therefore not surprising that many biogeographical studies have revealed a cardinal influence of temperature on marine biodiversity[[12-15](#_ENREF_12)].

However, we are aware that multiple environmental parameters influence the productivity and distributions of individual marine species, and their grouping together as communities and ecosystems[[16](#_ENREF_16)]. Nutrients and light limit phytoplankton production[[17](#_ENREF_17), [18](#_ENREF_18)]. Both bathymetry and local spatial variability in bathymetry are key determinant of the marine pelagic biodiversity[[19](#_ENREF_19)]. Dissolved oxygen must remain high enough to support respiration[[20](#_ENREF_20)]. Mixed Layer Depth (MLD) is an important parameter for phytoplankton production and controls the spatial distribution of many plankton species[[17](#_ENREF_17), [21](#_ENREF_21)]. Oceanic pH influences calcifying organisms such as coccolithophorids, foraminifers, corals and pteropods[[22](#_ENREF_22), [23](#_ENREF_23)]. Wind intensity affects prey-predator encounter rates[[24](#_ENREF_24)] by its effects on oceanic turbulence, and nutrient supply rates by its effects on vertical mixing[[21](#_ENREF_21)]. Wind direction, by its control of the distribution of some meroplankton species, might strongly affect recruitment of some benthic organisms [[25](#_ENREF_25)]. The above list is far from exhaustive, but shows the complexity of pathways and types of control that the environment might exert on organisms and biocoenoses.

Future version of our framework should therefore consider several environmental parameters simultaneously. However, this may not improve significantly the model because many environmental parameters covary with temperature (e.g. ice, oxygen, some nutrients). Furthermore, many environmental parameters are not available with sufficient accuracy on a global scale and on a year-to-year basis. That is why we have first focused on temperature.

Our model cannot realistically implement biotic interactions in the construction of pseudo-communities on a global scale. Multiple examples suggest that they can be quite important in some ecosystems[[9](#_ENREF_9), [26](#_ENREF_26)], however.

**References**

1. Hutchinson GE. An introduction to population ecology. New Haven: Yale University Press; 1978. 260 p.

2. Stevens GS. The latitudinal gradient in geographic range: how so many species coexist in the tropics. The American Naturalist. 1989;133:240-56.

3. Beaugrand G, Edwards M, Brander K, Luczak C, Ibañez F. Causes and projections of abrupt climate-driven ecosystem shifts in the North Atlantic. Ecology Letters. 2008;11:1157-68.

4. Sunday JM, Bates AE, Dulvy NK. Thermal tolerance and the global redistribution of animals. Nature Climate Change. 2012:1-5. doi: 10.1038/NCLIMATE1539.

5. Pörtner HO, Farrell AP. Physiology and climate change. Science. 2008;322:690-2.

6. Brown JH, Gillooly JF, Allen AP, Savage VM, West GB. Toward a metabolic theory of ecology. Ecology. 2004;85:1771-89.

7. Beaugrand G, Reid PC, Ibañez F, Lindley JA, Edwards M. Reorganisation of North Atlantic marine copepod biodiversity and climate. Science. 2002;296:1692-4.

8. Schmidt-Nielsen K. Animal physiology: adaptation and environment. 4 ed. New York: Cambridge University Press; 1990. 602 p.

9. Kirby RR, Beaugrand G. Trophic amplification of climate warming. Proceedings of the Royal Society London B: Biological Sciences. 2009;276:4095–103.

10. Sarmiento JL, Slater R, Barber R, Bopp L, Doney SC, Hirst AC, et al. Response of ocean ecosystems to climate warming. Global Biogeochemical Cycles. 2004;18:1-23. doi: GB3003, doi: 10.1029/2003GB002134.

11. Beaugrand G, Edwards M, Legendre L. Marine biodiversity, ecosystem functioning and the carbon cycles. Proceedings of the National Academy of Sciences of the USA. 2010;107:10120-4.

12. Rutherford S, D'Hondt S, Prell W. Environmental controls on the geographic distribution of zooplankton diversity. Nature. 1999;400:749-53.

13. Rombouts I, Beaugrand G, Ibañez F, Gasparini S, Chiba S, Legendre L. Global latitudinal variations in marine copepod diversity and environmental factors. Proceedings of the Royal Society B. 2009;276:3053-62.

14. Rombouts I, Beaugrand G, Ibañez F, Gasparini S, Chiba S, Legendre L. A multivariate approach to large-scale variation in marine planktonic copepod diversity and its environmental correlates. Limnology and Oceanography. 2010;55:2219-29.

15. Sunagawa S, Coelho LP, Chaffron S, Kultima JR, Labadie K, Salazar G, et al. Structure and function of the global ocean microbiome. Science. 2015;348(6237). doi: 10.1126/science.1261359.

16. Cloern JE, Jassby AD. Drivers of change in estuarine-coastal ecosystems: discoveries from four decades of study in San Francisco Bay. Reviews of Geophysics. 2012;50:rg4001. doi: 10.1029/2012RG000397.

17. Sverdrup HU. On conditions for the vernal blooming of phytoplankton. Journal du Conseil Permanent International pour l'Exploitation de la Mer. 1953;18:287-95.

18. Behrenfeld MJ. Abandoning Sverdrup's critical depth hypothesis on phytoplankton blooms. Ecology. 2010;91:977-89.

19. Helaouët P, Beaugrand G. Macroecology of *Calanus finmarchicus* and *C. helgolandicus* in the North Atlantic Ocean and adjacent seas. Marine Ecology Progress Series. 2007;345:147-65.

20. Goberville E, Beaugrand G, Sautour B, Tréguer P. Climate-driven changes in coastal marine systems of Western Europe. Marine Ecology Progress Series. 2010;408:129:47.

21. Longhurst A. Ecological geography of the sea. Amsterdam: Elsevier; 2007. 542 p.

22. Orr JC, Fabry VJ, Aumont O, Bopp L, Doney SC, Feely RA, et al. Anthropogenic ocean acidification over the twenty-first century and its impact on calcifying organisms. Nature. 2005;437:681-6.

23. Kroeker KJ, Kordas RL, Crim RN, Singh GG. Meta-analysis reveals negative yet variable effects of ocean acidification on marine organisms. Ecology Letters. 2010;13:1419-34.

24. Rothschild BJ, Osborn TR. Small-scale turbulence and plankton contact rates. Journal of Plankton Research. 1988;10:465-74.

25. Jolly MT, Guyard P, Ellien C, Gentil F, Viard F, Thiébaut E, et al. Population genetics and hydrodynamic modeling of larval dispersal dissociate contemporary patterns of connectivity from historical expansion into European shelf seas in the polychaete *Pectinaria koreni* (Malmgren). Limnology and Oceanography. 2009;54:2089-106.

26. Estes JA, Terborgh J, Brashares JS, Power ME, Berger J, Bond WJ, et al. Trophic downgrading of Planet Earth. Science. 2011;333:301-6.
