# Supplementary material for: Marine biodiversity and the chessboard of life
Source: PLoS One. 2018 Mar 22;13(3):e0194006. doi: 10.1371/journal.pone.0194006 (PMC5864006; doi:10.1371/journal.pone.0194006)
Supplement: S3 Text — (DOCX) [file pone.0194006.s008.docx]

**S3 Text | Correction of the estimations of species richness for all taxonomic groups**

***Protozooplankton***

For protozooplankton, we used two different estimates of the maximum number of species remaining to be discovered.

The first estimate was based on the assumption that 75% remain to be named (percentage corresponding to Protozoa and some Chromista in Appeltans and colleagues[[1](#_ENREF_1)]): 75% of 1350 already known species (their Table 2): this gave 1013 species. Therefore w (i.e. the total number of species in the ecological guild or functional type; here protozooplankton) was fixed to 1350+1013=2363 species in Equations (8) and (10) (see main text). As a result, z (i.e. the total number of species in all ecological guilds or functional types) was fixed to 8035+1013=9048 species in equations (9) and (10).

The second estimate was based on the recent assessment of plankton biodiversity made during TARA expedition[[2](#_ENREF_2)]. By analyzing 18S ribosomal plankton size-fractionated samples, eukaryotic ribosomal diversity was assessed to be ~150000 operational taxonomic units (OTUs). Protists accounted for at least 85% of all OTUs, be 127500 OTUs. Because on the 5700 protists already described, 4350 species belong to phytoplankton and 1350 to protozooplankton, we considered that 23.7% of the 127500 protists may be protozooplankton: this gave 30192 species. Therefore, the total number of species to be named was 30192 -1350 = 28842. This estimate was conservative because many species can be mixotrophic, from autotrophy to heterotrophy. An increase in this number would increase saturation rates. Therefore w was fixed to 30192 species in Equations (8) and (10) (see main text). As a result, z was fixed to 8035+28842=36877 species in equations (9) and (10).

***Metazooplankton***

For metazooplankton, we also used two different estimates of the maximum number of species remaining to be discovered.

The first estimate was calculated as follows:

1. **Copepods**. There are currently 10000 known marine copepods[[1](#_ENREF_1)](their Table 2), of which 2200 are pelagic[[3](#_ENREF_3)]. Therefore, they represent a large fraction of metazooplankton. Because 17-25% of marine copepods are currently known[[1](#_ENREF_1)] (their Table 2), we assumed that a maximum of 83% of 2200 species remains to be described: this gave 1826 species.
2. **Gelatineous plankton**. They also represent an important metazooplantonic group. We based our estimations of the species remaining to be discovered on a few key groups: Scyphozoa (201 species, 45.5% of species remaining to be described), Ctenophora (190 species, 49% of species remaining to be described), Larvacea (67 species, 50% of species remaining to be described), Thaliacea (79 species, 24% of species remaining to be described)[[1](#_ENREF_1)](their Table 2). For those 537 species, we assumed that 237 species remained to be described.
3. **Other metazooplankton** (2763 species). For the remaining species, crustaceans (e.g. some mysids, ostracods, cumaceans, cladocera), gelatinous plankton (e.g. jellyfish and siphonophores), arrow worms, pteropods, planktonic polychaetes, we assumed that the maximum of species remaining to be discovered was 50%, a maximum rate based on Appeltans and colleagues[[1](#_ENREF_1)](their Table 2) and Beaugrand[[3](#_ENREF_3)]: 1382 species.

Therefore, we assumed that 1826+237+1382=3445 species remaining to be discovered for metazooplankton. Therefore w was fixed to 5500+3445=8945 species in Equations (8) and (10) (see main text). As a result, z was fixed to 8035+3445=11480 species in equations (9) and (10).

The second estimate, also based on the recent assessment of plankton biodiversity made during TARA expedition[[2](#_ENREF_2)], was based on the fact that 15% of all assessed OTUs were metazooplankton: 15% of 150000 =22500 species. Therefore from this estimate, the total number of species remaining to be named is 22500-5500=17000. Therefore w was fixed to 22500 species in Equations (8) and (10) (see main text). As a result, z was fixed to 8035+17000=25035 species in equations (9) and (10).

***Fish***

For pelagic fish, we estimated the maximum number of species remaining to be discovered was 23% (percentage corresponding to Protozoa and some Chromista in Appeltans and colleagues[[1](#_ENREF_1)] (their Table 2)): 23% of 1068 species: 246 species. Therefore w was fixed to 1068+246=1314 species in Equations (8) and (10) (see main text). As a result, z was fixed to 8035+246=8281 species in equations (9) and (10).

***Marine mammals***

For marine mammals, Appeltans and colleagues[[1](#_ENREF_1)] (their Table 2) estimated that the maximum number of species remaining to be discovered was 8 species (only cetaceans). Therefore w was fixed to 117+8=125 species in Equations (8) and (10) (see main text). As a result, z was fixed to 8035+8=8043 species in equations (9) and (10).

**References**

1. Appeltans W, Ahyong Shane T, Anderson G, Angel Martin V, Artois T, Bailly N, et al. The magnitude of global marine species biodiversity. Current Biology. 2012;22:2189-202.

2. de Vargas C, Audic S, Henry N, Decelle J, Mahé F, Logares R, et al. Eukaryotic plankton diversity in the sunlit ocean. Science. 2015;348(6237). doi: 10.1126/science.1261605.

3. Beaugrand G. Marine biodiversity, climatic variability and global change. Oceans E, editor. London: Routledge; 2015. 474 p.
