# Supplementary material for: Marine biodiversity and the chessboard of life
Source: PLoS One. 2018 Mar 22;13(3):e0194006. doi: 10.1371/journal.pone.0194006 (PMC5864006; doi:10.1371/journal.pone.0194006)
Supplement: S4 Text — (DOCX) [file pone.0194006.s009.docx]

**S4 Text | Assessment of the mean thermal optimum and range of all taxonomic groups**

We assessed the mean thermal optimum and range of all taxonomic groups to examine potential relationships with niche saturation (Fig. 1; Methods). Fish had thermal optima (26.65-27.85°C) higher than plankton (22.65-24.45°C) and marine mammals (-0.05-17.15°C). Cetaceans had a degree of mean thermal range (0.84) higher than plankton (0.61-0.65) and fish (0.39-0.50). Pinnipeds had the smallest mean thermal range (0.17) and optimum (-0.05°C), which can probably be explained by the relatively young monophyletic origin of the taxonomic group [[1](#_ENREF_1)].

**References**

1. Arnason U, Gullberg A, Janke A, Kullberg M, Lehman N, Petrov EA, et al. Pinniped phylogeny and a new hypothesis for their origin and dispersal. Molecular Phylogenetics and Evolution. 2006;41:345-54.
